# Tailored Polyelectrolyte Multilayer Systems by Variation of Polyelectrolyte Composition and EDC/NHS Cross-Linking: Physicochemical Characterization and In Vitro Evaluation

**DOI:** 10.3390/nano12122054

**Published:** 2022-06-15

**Authors:** Uwe Schirmer, Johanna Ludolph, Holger Rothe, Nicole Hauptmann, Christina Behrens, Eva Bittrich, Henning Schliephake, Klaus Liefeith

**Affiliations:** 1 Institute for Bioprocessing and Analytical Measurement Techniques, 37308 Heiligenstadt, Germany; uwe.schirmer@iba-heiligenstadt.de (U.S.); johanna.ludolph@t-online.de (J.L.); holger.rothe@iba-heiligenstadt.de (H.R.); nicole.hauptmann@iba-heiligenstadt.de (N.H.); 2 Department of Oral and Maxillofacial Surgery, George-Augusta-University, 37075 Goettingen, Germany; cbehren@gwdg.de (C.B.); schliephake.henning@med.uni-goettingen.de (H.S.); 3 Center Macromolecular Structure Analysis, Leibniz Institute of Polymer Research, 01005 Dresden, Germany; bittrich-eva@ipfdd.de

**Keywords:** polyelectrolyte multilayer (PEM), EDC/NHS cross-linking, collagen immobilization, bone implants, PEM surface characterization, PEM biocompatibility

## Abstract

The layer-by-layer (LbL) self-assembly technique is an effective method to immobilize components of the extracellular matrix (ECM) such as collagen and heparin onto, e.g., implant surfaces/medical devices with the aim of forming polyelectrolyte multilayers (PEMs). Increasing evidence even suggests that cross-linking influences the physicochemical character of PEM films since mechanical cues inherent to the substrate may be as important as its chemical nature to influence the cellular behavior. In this study, for the first-time different collagen/heparin films have been prepared and cross-linked with EDC/NHS chemistry. Quartz crystal microbalance, zeta potential analyzer, diffuse reflectance Fourier transform infrared spectroscopy, atomic force microscopy and ellipsometry were used to characterize film growth, stiffness, and topography of different film systems. The analysis of all data proves a nearly linear film growth for all PEM systems, the efficacy of cross-linking and the corresponding changes in the film rigidity after cross-linking and an appropriate surface topography. Furthermore, preliminary cell culture experiments illustrated those cellular processes correlate roughly with the quantity of newly created covalent amide bonds. This allows a precise adjustment of the physicochemical properties of the selected film architecture regarding the desired application and target cells. It could be shown that collagen improves the biocompatibility of heparin containing PEMs and due to their ECM-analogue nature both molecules are ideal candidates intended to be used for any biomedical application with a certain preference to improve the performance of bone implants or bone augmentation strategies.

## 1. Introduction

Despite the fact that the formation of polyelectrolyte multilayers (PEMs) has been the focus of numerous research activities for decades, the investigations regarding specific applications to control the surface composition and the properties of biomaterials that may decide about the success or failure of medical interventions are still increasing. The possibility for the simple generation of artificial ECMs with natural polyelectrolytes such as charged polysaccharides and proteins might be one reason for the growing interest in using polyelectrolytes in life science applications [1,2,3]. A well-known procedure for the deposition of polyelectrolytes on substrates is the “Layer by Layer” (LbL) assembly of polyelectrolytes. Among a variety of methods for surface modifications, this technique has emerged as a quite simple, cost-efficient, and versatile option to fabricate thin biomimetic films. Beside several other possible applications, any such biomimetic films are more and more in the focus of investigations for the improvement of implant materials used in the field of bone replacement, bone augmentation, and bone tissue engineering [1,4,5,6]. However, it is well accepted that for the generation of valuable osseointegrative implants several factors should be considered. This includes the PEM composition to mimic the site-specific extra cellular matrix (ECM), physicochemical properties as well as the integration of cytokines that contribute to bone tissue regeneration. ECM molecules such as glycosaminoglycan’s (GAGs) (e.g., chondroitinsulfate (CS), heparin (Hep) and hyaluronic acid (HA)) and fibrillary glycoproteins such as collagens, fibronectins, and laminins are becoming more and more in focus for the preparation of bioactive surfaces that mimic the native ECM [7]. Hence, the deposition of ECM molecules by LbL is an excellent option to establish an ECM-like microenvironment and to improve the osseointegration of implants [8,9,10,11]. Natural polymers such as collagen, fibronectin, vitronectin, or laminin have the great advantage that they already contain specific binding motives like GFOGER, RGD, SIKVAV, or YIGSR that enable cell adhesion, proliferation, and differentiation in combination with synthetic implant materials [12]. Collagen type I is the most common protein in the ECM with a defined 3D structure that also contains specific cell binding sites. Hence, collagen defines not only the physicochemical properties of the ECM and the tissue but also supports the direct adhesion as well as the migration of cells via intrinsic receptor molecules. Furthermore, it is described that collagen type I matrices exhibit a tremendous binding capacity for distinct molecules like growth factors and cytokines [13]. However, independent of the tissue type, the ECM is composed of up to 300 different core proteins and up to 200 GAGs [14]. Among the group of GAGs, a recent analysis reveals that heparin has more protein interaction capacity than any other GAG or ECM molecule [15] and is therefore a relatively good reservoir for cytokines [16,17].

There is no doubt that the biocompatibility as well as the biofunctionality of bone implant materials can be strongly influenced by the chemical composition and the physical properties, such as wettability, surface charge, and topography in order to affect initial protein adsorption, cell behavior, and ultimately osteointegration and bone tissue regeneration. It is known that cell programming can also be influenced by physical and chemical cues incorporated in material surfaces [18,19,20]. Indeed, the differentiation of osteoblast progenitor cells correlates with the stiffness of the substrate and is strongly inhibited on soft materials [19,21]. Chemical cross-linking can influence the physiochemical properties of an established PEM. Different methods have been reviewed by Ghiorghita and Qi An [22,23]. A well investigated and established method for film cross-linking is the chemical reaction with EDC (1-ethyl-3-(3-dimethylaminopropyl)carbodiimide) and NHS (N-hydroxysuccinimide). The carbodiimide-reaction catalyzes the formation of amide bonds from the carboxylic and amine groups of the reaction partners. Within the amino acid sequence of collagen both functional groups can be found, whereas in the polypeptide poly-L-lysine (PLL) only amino groups and in the GAG heparin only carboxyl groups are available. In the meantime, EDC/NHS chemistry is a commonly accepted tool in cell biological applications. Several investigations confirm the functionality of EDC-modified PLL/HA multilayers in order to control the stiffness by means of different degrees of cross-linking [24] and the appropriate response of different cell types like chondrocytes [25], endothelial cells [26], human mesenchymal stem cells [27] or myoblasts [18]. Furthermore, EDC/NHS changes the release of bioactive compounds like BMP2 [28].

Hence, the preparation of polyelectrolyte multilayers to produce bioactive coatings has been recently emerged as an alternative route to deliver various cellular stimuli: (i) the availability of cell activating motifs, (ii) their biomechanical properties, and (iii) their functionality as cytokine reservoir that influences the cellular fate.

These characteristics should be considered when creating a PEM system for a specific application in a specific tissue. To achieve the most valuable effect regarding biofunctionality and tissue integration, the PEM system should be adaptable to fit the physiological situation and requirements of the target tissue. Thus, it was the motivation of the authors to generate a simple and highly flexible collagen–heparin-based PEM platform that allows the variation of chemical composition, thickness, and stiffness under well-defined cross-linking conditions. Thus, it is of particular interest how the PEM composition and concentration-dependent cross-linking affects the cell response. To the best of our knowledge only a handful of papers describe the stepwise multilayer formation and the biocompatibility of PLL-Hep PEMs and no publication exists that describes collagen–Hep systems. Consequently, it can be assumed that these novel bioactive PEM films will form the basis for numerous highly specific functional coatings in order to meet the requirements of numerous biomedical applications.

## 2. Materials and Methods

### 2.1. Multilayer Film Preparation and Cross-Linking

Chemicals and reagents were used without further purification, unless stated otherwise. PEM films were assembled from poly-L-lysine (PLL) (Merck, Darmstadt, Germany P2636, PLL, 30–70 kDa), heparin (Hep) (Merck, Darmstadt, Germany, H3393, heparin from porcine intestinal mucosa, 6–30 kDA), and collagen (Col) (ibidi, Graefelfing, Germany 50202, rat tail collagen type I, 10 mg/mL). The polyelectrolytes for (PLL-Hep) films were dissolved in Na-acetate buffer (20 mM, pH 4.5) at a concentration of 1 mg/mL. For deposition of (Col-Hep) multilayers the polyelectrolytes were dissolved in 5 mM Acetat (pH 3.5) at a concentration of 1 mg/mL. Film construction was performed automatically employing a dipping robot (DR3, Riegler&Kirstein, Potsdam, Germany). Briefly, the cleaned substrates were first soaked into the polycation solution (PLL) and left to adsorb for 5 min. Subsequently, the samples were soaked into three deionized water wash solutions to rinse the surface and to remove unbound polyelectrolytes. Subsequently, Hep was deposited in the same manner. For (Col-Hep) film construction the substrate was dipped for 30 min into the solved polyelectrolyte. The washing was the same as described for (PLL-Hep) films. Each cycle was repeated until reaching the desired number of double layers. All samples were rinsed in deionized water and air dried in a gentle stream of pressurized air.

The following cross-linking was done using EDC/NHS (abcr, Karlsruhe, Germany, EDC—AB181824, NHS—AB110699) at five EDC concentration levels (0, 5, 25, 100, 200 mg/mL). The EDC stock solution (400 mg/mL) was prepared in ice cold 0,15 M NaCl, pH 5 and from this further diluted in separate volumes until two-fold concentrations from each target concentration was reached. Finally, these two-fold pre-dilutions were further mixed in an equal volume with a 22 mg/mL NHS solution. Each cross-linking reaction was done in 1 mL EDC/NHS-solution in 24-well plates. All working steps were performed on ice with pre-cooled buffer and all samples were incubated overnight at 4 °C. After the cross-linking, all samples were washed at room temperature three times with 0.15 M NaCl, pH 8. Between each washing step, the samples were incubated for 1 h. Finally, the samples were rinsed with water and allowed to air-dry at room temperature.

### 2.2. Films Characterization by Quartz Crystal Microbalance with Dissipation Monitoring (QCM-D)

The measurements were performed by using the QCM system from Q-Sense (BiolinScientific, Gothenburg, Sweden). The quartz crystal (QS-QSX336, BiolinScientific, Gothenburg, Sweden) was excited at its fundamental frequency (about 5 MHz, ν = 1) as well as at the third, fifth, and seventh overtones (ν = 3, 5 and, 7 corresponding to 15, 25, and 35 MHz, respectively). Beside changes in the resonance frequencies ∆f, also the relaxation of oscillation after crystal excitation ∆D (dissipation) was detected. The deposition of the layers occurred under permanent flow (0.2 m l/min) for 10 min. Each polyelectrolyte was diluted in the same buffer as used for the film preparation by dipping. After binding of the polyelectrolyte by charge compensation, pure buffer was pumped through the flow cell for 5 min. After deposition of a complete double layer, the flow was stopped for five more minutes. After this, the next cycle of double layer deposition started again. The number of cycles were repeated until the desired number of double layers was reached. For analysis of polyelectrolyte binding shifts, ∆f and ∆D were continuously recorded over the whole film deposition process. Film thickness was modeled with the Dfind software (BiolinScientific, Gothenburg, Sweden) from Q-Sense using a viscoelastic Voigt model in which the adsorbed film is represented by a (frequency dependent) complex shear modulus.

### 2.3. Cross-Linking Analysis by FTIR

For film cross-linking characterization, the relevant cross-linked films deposited on a ZnSe crystal were investigated by in situ Fourier transform infrared spectroscopy (FTIR) in attenuated total reflection (ATR) mode with an Equinox 55 spectrophotometer (Bruker, Wissembourg, France). For preparation and measuring of cross-linked films, D_2_O instead of H_2_O was used as the solvent. This reduces the water band absorption around 1643 cm^−1^, which also affects the amide I bands of both PLL and heparin. However, all films were prepared as described above. After film deposition, single-channel spectra from 512 interferograms were recorded between 400 and 4000 cm^−1^ with a 2 cm^−1^ resolution, using Blackman–Harris three-term apodization and the standard Bruker OPUS/IR software (Version 3.0.4, Bruker, Wissembourg, France). Finally, the intensity of amide I and amide II bands were analyzed.

### 2.4. AFM Measurements

All AFM measurements were carried out according to a method we published elsewhere, using a JPK instruments AG Nanowizard IV Bio-AFM (Bruker Nano GmbH, Berlin, Germany) equipped with an CellHesion extension for a z-movement of up to 100 µm [29]. Measurements were performed in aqueous solution taking the swollen state of the PEM films into consideration. Glass spheres (d = 11.5 μm) fixed at the end of Nanoworld™ Arrow TL-1 Cantilevers (Nanoworld, Neuchâtel, Switzerland) were used for indentation. Force distant curves were obtained with a final z-length of 5 µm, an indentation speed of 1 µm s-1 and a sampling rate of 5 kHz. Indentations were performed on areas of (25 × 25) µm^2^ with a grid of 12 × 12 single measurements. On each sample three independent areas were scanned and for each coating three independent samples were investigated.

Young’s moduli were calculated based on the approach curves by implementing the Hertzian contact model for spherical geometries. To exclude substrate effects the first 20 nm of indentation were used for calculation, which represents ca. 10% of film thickness [29].

### 2.5. Thickness and Topography Analysis with In Situ-Ellipsometry

The common ellipsometric angles delta (∆) and psi (Ψ) were recorded with an imaging ellipsometer (EP3, Accurion, Goettingen, Germany) equipped with both a 532 nm laser and a full spectrum lamp (Xenon Short ARC^®^, Osram, Munich, Germany). PEM film systems were deposited on SiO_2_ wafers by dipping as described above. PEM film thicknesses on silicon (SiO_2_: n = 1.455, k = 0.00, d = 3 nm, Si: n = 4.16, k = 0.049) were recorded with up to 15 distinct wavelengths between 351 nm and 890 nm with an angle of incidence of 54°. For imaging measurements ∆ and Ψ maps of (529 × 401) µm^2^ were recorded with a 5-fold magnification objective. All measurements were performed in double-distilled water since this was also the rinsing buffer for the film build-up. The in situ cell was a self-made 3D printed cell with optical transparent sight windows with an angle of 54°. The obtained angles were transformed into film thicknesses by employing a fixed n and k model implemented within the software. Thickness was modeled (EP4Model software 1.2.0, Accurion, Goettingen, Germany) with a Cauchy–Urbach model due to a tail of absorption band in l-lysine at wavelengths below 400 nm (PEM1 and PEM2: n (601) = 1.336; PEM3: n (601) = 1.332; k). In order to obtain topography mapping a separate image at 601 nm wavelength was taken. Based on Ψ values for each point, thickness values were calculated with the same parameters and the model described above. For the mapping, the single point mode of the mapping tool was used. Thus, a topography map based on thickness data for each single image pixel was created. The grey-scale image was imported to the SPIP software (SPIP 6.6.5, Image Metrology A/S, Lyngby, Denmark) and further processed. Before roughness analysis an ISO16610 Gaussian L-filter according to the ISO 25178-2 standard was applied. In this case, the filter eliminated 1/7th of all large wavelength components.

### 2.6. Zeta Potential Measurements

Zeta potential measurements were employed to investigate the charge compensation after each coating step. For this purpose, the manually deposited individual layers of polycations and polyanions were incubated for 5 min each. After coating, the samples were washed and the zeta potential was measured threefold in 1 mM NaCl at pH 5.5. All measurements were performed using the Surpass-2 (Anton Paar GmbH, Graz, Austria) equipped with a micro-slit measurement unit. A slit of 120 µm was set and the measurement of the streaming potential was performed at 300 mbar.

### 2.7. Cell Culture Experiments

All PEM coatings for cell biological analyses were prepared on glass discs and sterilized by UV irradiation. MC3T3-E1 cells (DSMZ, Braunschweig, Germany) were cultivated with α-MEM medium (P04-21500) with 10% fetal bovine serum (*v*/*v*) and 1% penicillin/streptomycin (*v*/*v*) (37 °C, 5% CO_2_, 80% humidity). Media and supplements were purchased from PAN-biotech (Aidenbach, Germany). Medium was exchanged every two to three days and for cell expansion or cell seeding, cells were enzymatically detached from the surface with 0.05% (*w*/*v*) trypsin/0.02% (*w*/*v*) EDTA and re-suspended in serum-free medium. Before each experiment cells were stained with trypan blue and counted with a cell counter (Logos biosystems, Anyang-si, South Korea). Cells were prepared for proliferation, adhesion, and spreading analysis. All cell biological experiments were conducted in triplicate in at least three separate experiments.

Cell proliferation was measured by the XTT assay. The assay was conducted according to manufacturer’s instructions (X6493, Thermo Fisher Scientific, Waltham, MA, USA). To analyze the influence of PEM films on cell proliferation, 9000 cells were seeded in a 24-well plate with a PEM coated B33 glass disc. As control the cells were also seeded on uncoated glass discs or directly on TCPS. Thereafter, the cells were incubated as indicated and the increase in cell number analyzed with XTT. After incubation, 100 µL of the medium was transferred to a well of a 96-well plate. The reduction of the XTT was measured with a microtiter plate reader (Biotek, Winooski, VT, USA).

For cell adhesion analysis, 300 µL of cell suspension was added to each well at a density of 1 × 10^6^ cells/mL (equivalent to 1.58 × 10^5^ cells/cm^2^ of well surface) in serum-free medium with or without 5 mM EDTA. After incubation at 37 °C/5% CO_2_ for 60 min, loosely bound cells were removed with 3 × 300 µL PBS wash cycles. Bound cells were fixed with 4% PFA for 20 min at RT and stained with DAPI. Subsequently images of the complete surfaces were visualized by laser scanning microscopy (LSM710, Zeiss, Jena, Germany). Cells were counted with the Arivis Vision4D software (Arivis, Rostock, Germany).

For spreading analysis, 300 μL of cell suspension at 2 × 104 cells/mL in culture medium was added to the substrates and incubated overnight at 37 °C/5% CO_2_. The cells were fixed by the addition of 37% (*w*/*v*) formaldehyde (final concentration 3.7%) directly into the cell media for 20 min at room temperature. The samples were washed in 3 × 300 μL PBS, fixed with 4% PFA for 20 min at RT and double stained with Hoechst33258 (Thermo Fisher Scientific, Waltham, MA, USA) and Phalloidin488 (MoBiTec, Goettingen, Germany). Cells were viewed using a Zeiss LSM710 as described above. From each sample at least five pictures were taken and the cell spreading area was calculated with the Arivis Vision4D software (Arivis, Rostock, Germany).

For simple analysis of morphology, cells on the samples were rinsed twice with PBS and then fixed with 4% PFA for 20 min. Cells were stained with Phalloidin590 (MoBiTec, Goettingen, Germany) and Hoechst33258 (Thermo Fisher Scientific, Waltham, MA, USA) according to manufacturer instruction and analyzed with the confocal laser scanning microscope LSM710 (Zeiss, Jena, Germany).

### 2.8. Statistical Analysis

All results are shown as mean values ± standard error deviation.

## 3. Results

### 3.1. Film Construction and Kinetics

**Scheme 1:**
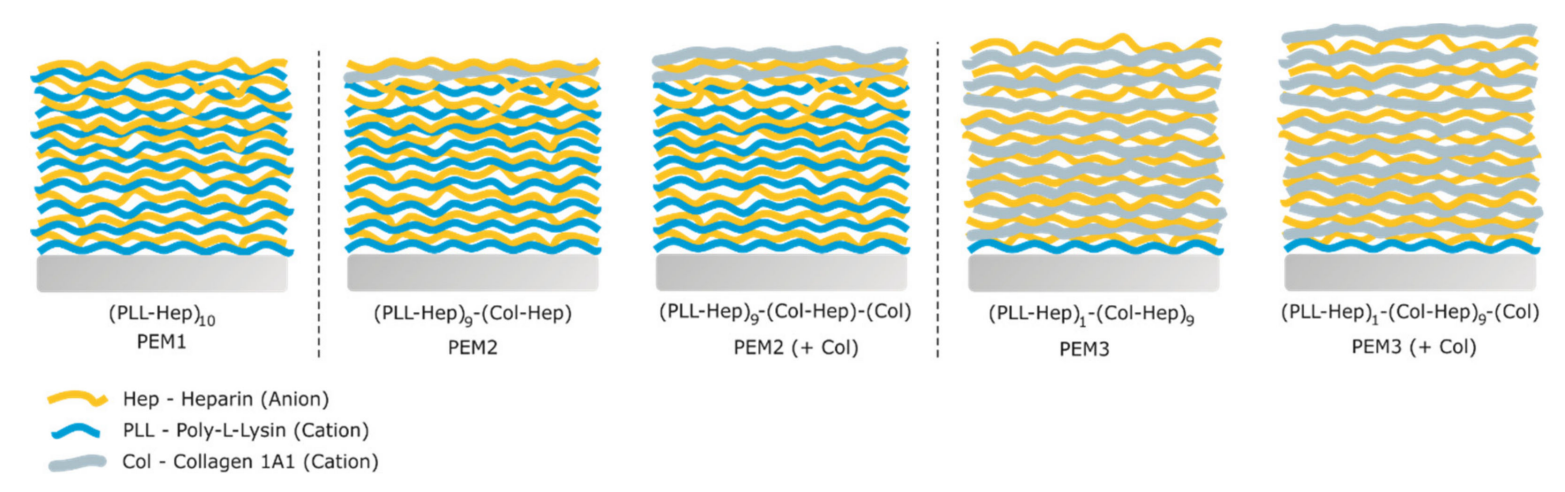
PEM1 was prepared by ten double layers of the well-established polyelectrolytes PLL and heparin. The polyelectrolytes PLL and Hep was also the basis for another PEM that was finally covered with a collagen layer. Hence, nine (PLL-Hep) double layers were followed by either one collagen-heparin double layer (PEM2) or in a variation by collagen-heparin-collagen (PEM2 + Col). In another PEM film, the PLL cation was in nine DL substituted by collagen (PEM3). For a better film, assembling the first DL was still prepared with PLL since collagen is, even under low pH, only a weak cation. As for PEM2, PEM3 films with a final collagen layer were prepared (PEM3 + Col).

To the best of our knowledge this is the first report that comprehensively describes the kinetics of PEM formation and the physicochemical characterization using the ECM molecules heparin and collagen as polyelectrolytes. Beside collagen containing films (PEM2 and PEM3), PEM films based on PLL-Hep were prepared as reference (PEM1). The designs of all generated PEM films are visualized schematically in Scheme 1. Overall, five different PEM films were prepared. The reference film (PLL-Hep)_10_, from here on referred to as PEM1, was composed from ten double layers of the cation PLL and the anion heparin. To achieve a better approximation of the physiological ECM compared with PEM1, two other film constructs were generated. In both PEMs, PLL was partially or completely substituted by collagen. Hence, in the so-called PEM2, collagen was deposited in the outermost double layer as a polycation, whereas in the third multilayer, herein referred to as PEM3, PLL was completely replaced by collagen except in the first double layer. In addition, for both systems a further PEM variation was established with an additional top layer made of collagen. Finally, all described PEMs were analyzed comprehensively by several methods.

**Figure 1:**
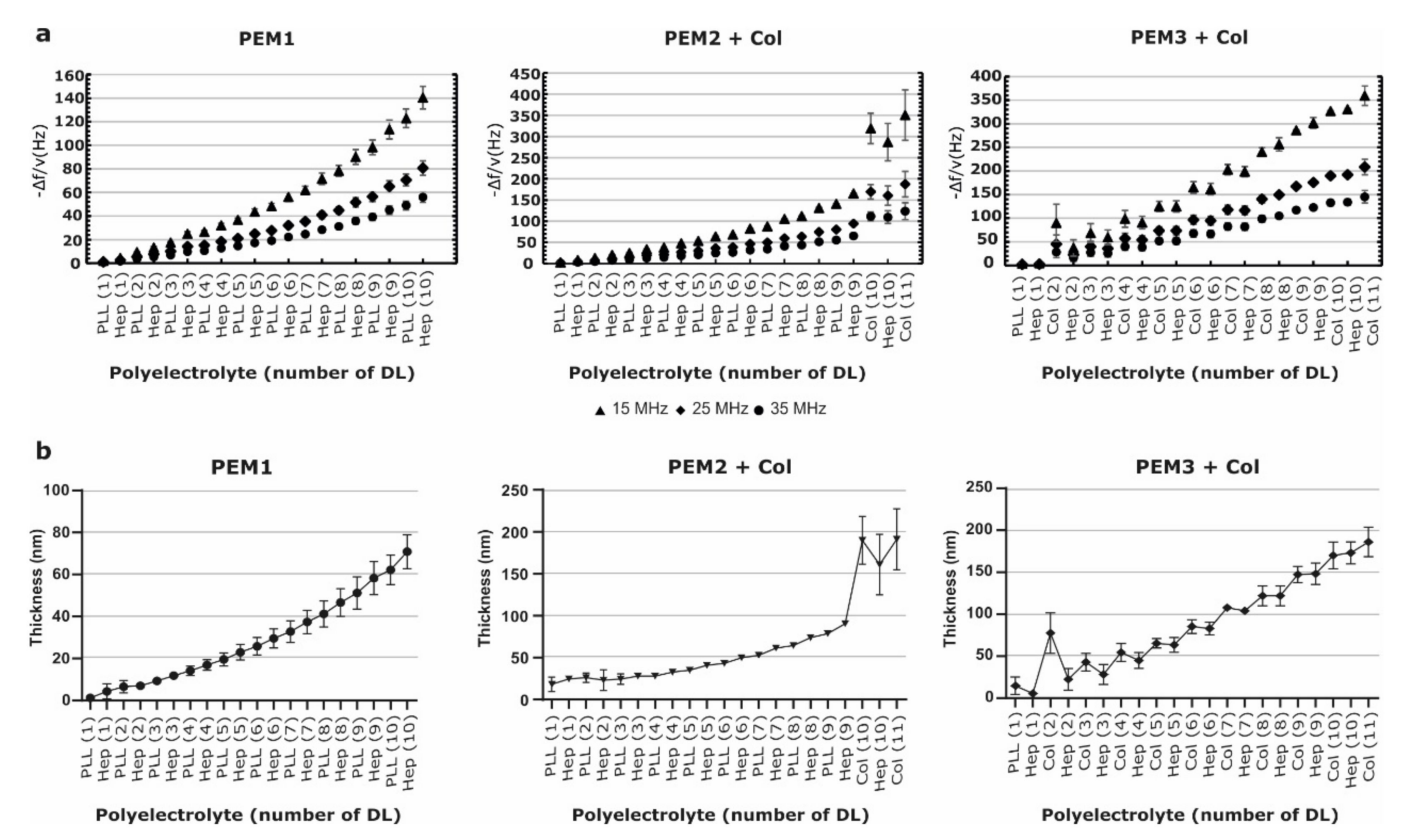
Online measurement of PEM film growth followed by in situ QCM-D. Shown are differences in the QCM (**a**) frequency (-∆f/v) and (**b**) thickness (nm) as a function of polyelectrolyte layer deposition. Brackets show the number of the accumulated bi-layers deposited on the QCM-D sensor. In (**a**) data are given for three harmonics: 15 MHz (ƒ); 25 MHz (F); and 35 MHz. Mean values are from at least three independent experiments. Each data point was calculated as mean from 10 values at the end of the deposition cycle. Each result was calculated from at least three single experiments.

The film growth was monitored by QCM-D and further characterized by zeta potential measurements. QCM-D data (Figure 1 and Figure S1) reveal an almost linear growth behavior for all investigated multilayer systems with a gradual transition to an exponential course. As expected, PEM1 and PEM2 show similar growth kinetics until the deposition of the first collagen layer as far as the PEM2 is concerned. For PLL and Hep polyelectrolytes, an almost continuous linear growth behavior until the deposition of the sixth double layer was observed. From the seventh double layer onwards, once again a weak exponential growth behavior could be detected. The exponential growth can be explained by the diffusion of free PLL molecules in the film and the increase of free PLL molecules that remain in the film with each new layer. Thus, with each new layer more anions can bind to the film [30]. However, it has to be stated that the non-linear growth is much lower in comparison with other PEMs with PLL as cation [31].

However, the exchange of the PLL by means of collagen at the cation position leads, obviously, to some discontinuities of the growth behavior. Thus, for PEM2 films a large frequency shift (∆f) of approximately 150 Hz was measured for the third overtone after collagen deposition in the outermost double layer (Figure S1). An additional deposition of collagen as the top layer of PEM2 led to a significantly lower frequency shift. Analogous observations about the growth behavior of the PEM3 film type reveals a similar effect. After the initial deposition of collagen in the second double layer, the ∆f value of the third overtone shifts clearly for almost 90 Hz compared with the base line, whereas the ∆f values are significantly reduced for the following collagen layers. However, for each new double layer upwards of the second double layer, a continuous film growth was monitored. The average of ∆f for the third overtone was about 36 Hz per double layer. The constant change in the frequency over all three overtones documents an approximately linear growth and no saturation could be seen on the sensor surface.

Nevertheless, in concordance with the frequency monitoring the polyelectrolyte multilayer formation shows a clear increase in the film thickness/mass after collagen deposition (Figure 1). Based on the measured frequency and dissipation data, multilayers with up to 200 nm thickness could be generated. It should be emphasized that despite the different numbers of (Col/Hep) double layers, both collagen containing film systems (PEM2/PEM3) show a comparable thickness of about 200 nm, whereas the collagen free (PLL/Hep) reference systems (PEM1) are, as previously described, only about 70 nm thick [32]. These results lead to the conclusion that the initial deposition of collagen is mainly responsible for the significant variation in thickness. However, it is also obvious that the PEM3 film thickness steadily increases after deposition of the first collagen layer. Thus, it can be further speculated that the initial adsorption process of the collagen is partially reversible since there is a massive loss of mass after the first washing step.

**Figure 2:**
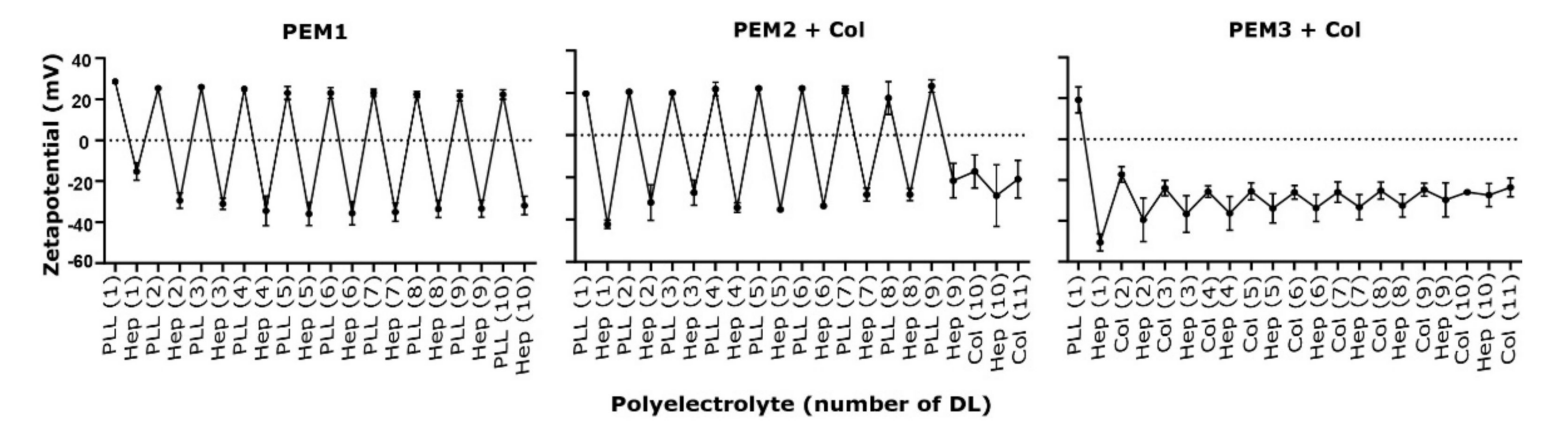
Change of the zeta potential for all three film systems as function of polyelectrolyte layer deposition. For each data point the mean values from three independent experiments are shown.

The results described so far referring to the differences in the growth behavior of the collagen-containing polyelectrolyte multilayers (Col/Hep) were confirmed by zeta potential measurements (Figure 2). It can be noticed that collagen is, as expected and previously described [32], only a weak cation in comparison to PLL and leads, obviously, to an incomplete intrinsic charge compensation. In Figure 2 it is shown that PLL inverts the negative surface charge induced by the anionic heparin. The ζ potential alternates from −40 mV to +20 mV after each PLL deposition. In contrast to PLL, the weak cationic collagen is not able to compensate the surface charge completely. Only a slight reversal of the surface charge was measured. Furthermore, the cationic effect of collagen becomes weaker with an increasing number of double layers.

### 3.2. Film Properties after Cross-Linking

It has long been known that physical cues such as surface topography or multilayer stiffness were also identified as key parameters to control cell behavior. Even when there are still some gaps about the exact mechanism by which cells transduce mechanical stimuli into a biochemical signal, increasing evidence suggests that mechanical cues are just as important as their biochemical counterparts. To this end, the described film systems were used for further cross-linking with EDC/NHS chemistry. The aim of the cross-linking is, on the one hand to generate PEM films with a tunable rigidity, but on the other hand to gain multilayer films with specific diffusion profiles and defined release properties. It is already well-described to use EDC/NHS chemistry for the cross-linking of PEM films [22]. Nevertheless, to the best of our knowledge, it has not been shown before that cross-linking of (PLL/Hep) or (Col/Hep) PEMs can be accomplished. Hence, nothing is known about the properties of such film types after cross-linking and how the properties can be tuned by cross-linking strategies.

The influence of EDC/NHS cross-linking on the different film types was investigated by Fourier transform infrared spectroscopy (FTIR), atomic force microscopy (AFM), zeta potential measurements, and ellipsometry. Each PEM system was treated with five different EDC concentrations. The aim was to generate PEM films with continually changing properties with increasing EDC concentrations, because one can assume that with increasing EDC concentration more ammonium groups are covalently cross-linked with corresponding carboxylate groups.

#### 3.2.1. Confirmation of the EDC/NHS Cross-Linking with FTIR Measurements

**Figure 3:**
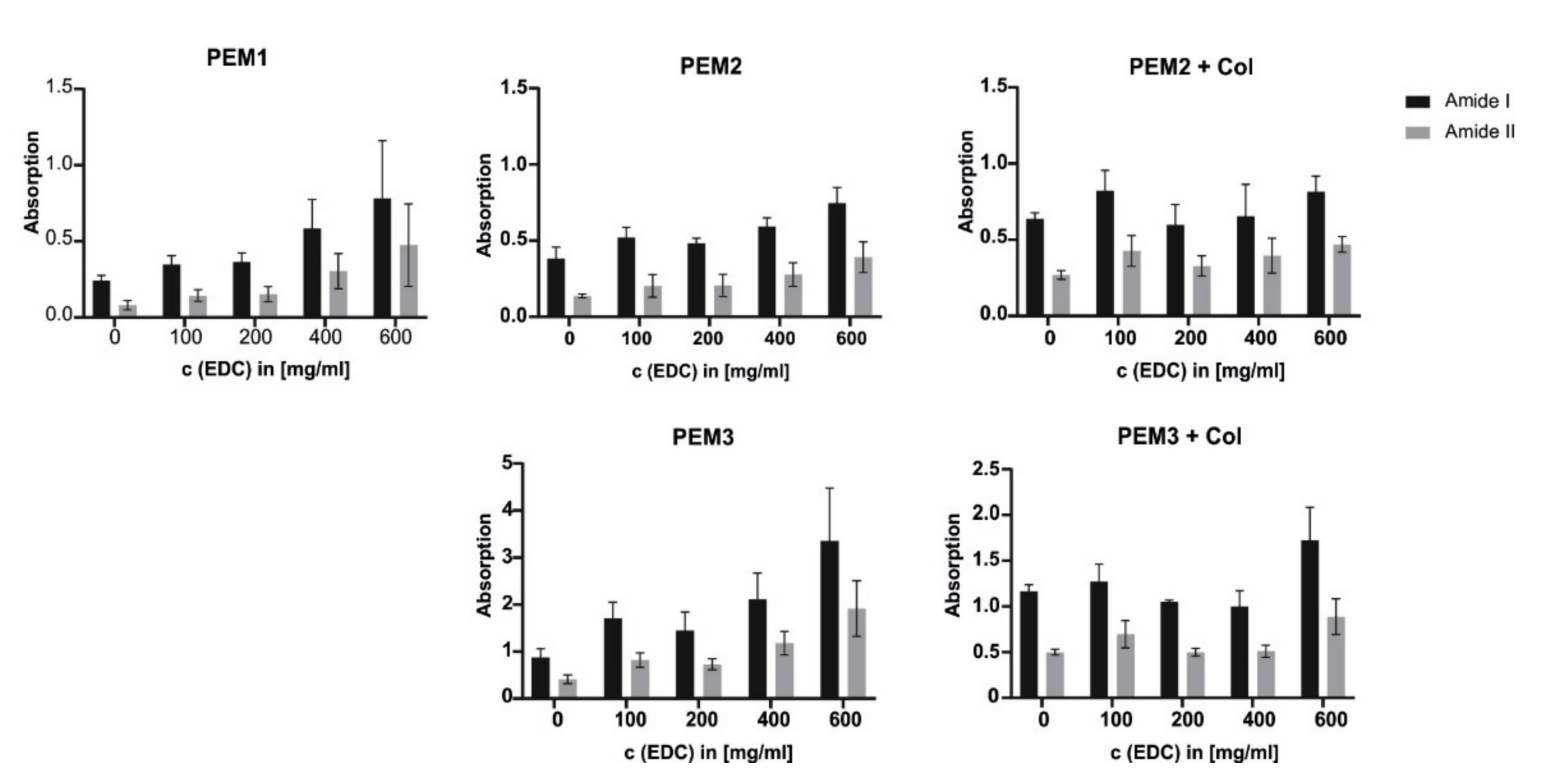
Absorption of ATF-FTIR spectra of native and cross-linked PEM films. The values (amid I and amid II) reflect the maximum from two characteristic peaks within the measured spectra. Each peak represents one specific amide bond formed within the film after contact with EDC. The height of the peaks correlates with the number of amide bonds.

This proposed effect was first examined with FTIR (Figure 3) following the increase in amide bonds that are formed by the reaction of primary ammonium groups with carboxylate groups. Indeed, two absorption peaks can be assigned to the newly created amide bonds. Amide I appears at 1690–1620 cm^−1^ and amide II at 1590–1520 cm^−1^ [33]. According to the proposed reaction mechanism, the intensity of absorption at these wavelengths should correlate with the EDC concentration. Indeed, for all analyzed PEM films a positive correlation of the absorption and the EDC concentration could be identified for both amide bonds. This finding indicates clearly that all PEM films are sensitive for EDC/NHS-treatment and that it is possible to introduce a specific number of new covalent bonds within the PEM films. EDC concentrations between 0 and 600 mg/mL have been chosen for the FTIR experiment and a linear increase of the absorption until the highest concentration could be observed for all PEM films.

#### 3.2.2. Cross-Linking Changes Viscoelasticity

**Figure 4:**
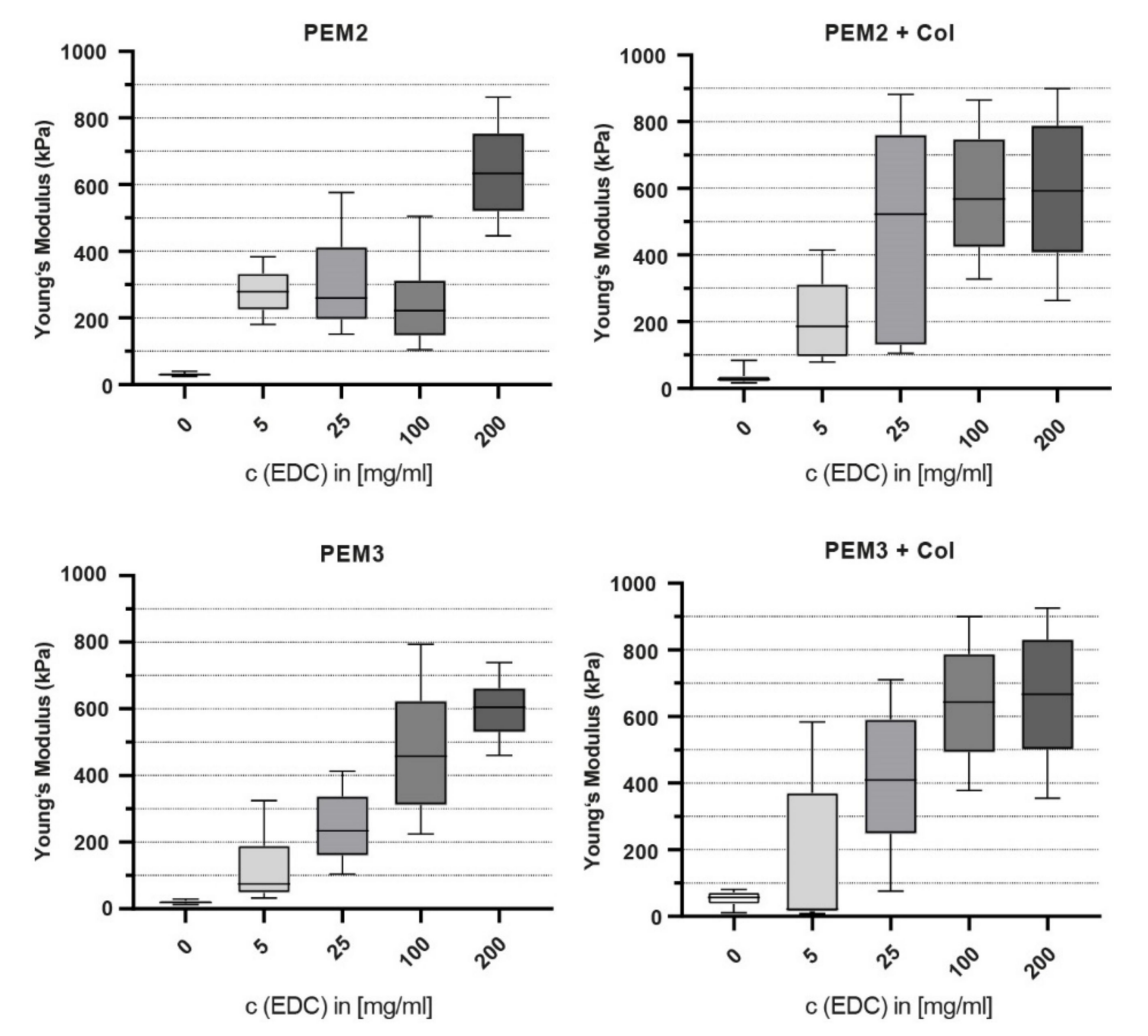
Atomic force microscopy (AFM) of cross-linked PEM films. Measurements were performed in aqueous solution taking the swollen state of the PEM films into consideration. From each sample three different areas with a dimension of (25 × 25) µm^2^ with a grid of 12 × 12 single measurements were measured. For the statistical analysis the measurements were conducted with three independent samples.

After confirmation of the sensitivity of PEM films for EDC/NHS chemistry, the EDC concentration was changed to lower concentrations between 0 and 200 mg to obtain PEM films with specific mechanical properties in a linear range. The measurement of the Young’s modulus of PEM films using colloidal AFM confirms the assumption that even lower EDC concentrations are suitable to tune the film properties regarding an appropriate viscoelasticity (Figure 4). Therefore, the overall tunable stiffness range was between 3 kPa and 650 kPa. With exception of PEM1, all multilayer systems could be analyzed via AFM. In case of PEM1 films the final thickness was too low to provide stiffness values independently from the underlying substrate. Interestingly, even at the lowest concentration of 5 mg/mL EDC, a significant increase in the stiffness was measured for all PEM2 and PEM3 film systems. The stiffness without any EDC treatment was in a range between 3 kPa and 40 kPa, whereas with 5 mg/mL the stiffness attains values around 100–250 kPa. Not surprisingly, the Young’s modulus was further increased at higher EDC concentrations and reaches maximum values of about 600–650 kPa.

#### 3.2.3. Thickness and Topography Analysis Performed by Ellipsometry

**Figure 5:**
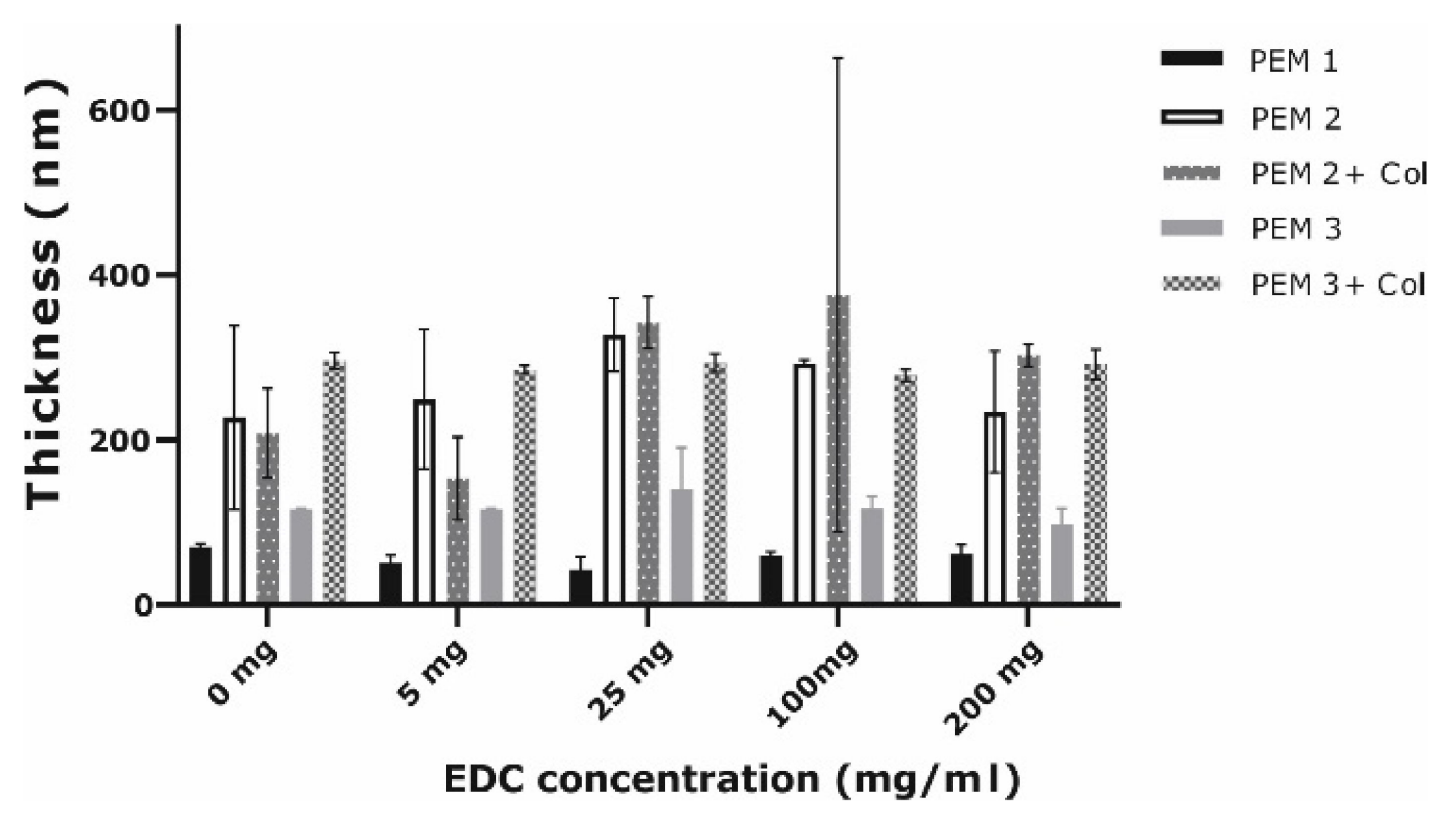
Film thickness from cross-linked film systems measured with ellipsometry. Data were obtained from three independent samples and three random areas for each sample. Film thickness was calculated with a Cauchy model.

**Figure 6:**
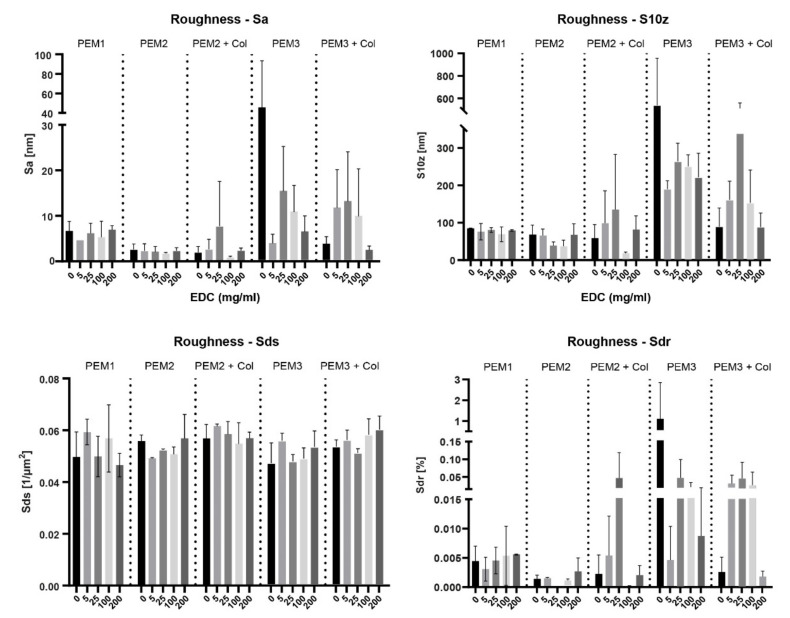
Roughness for EDC cross-linked PEM films. Images for the calculation of the different roughnesses were obtained from mapping with ellipsometry. Data were obtained from three independent samples and three random areas for each sample. Delta and psi values from each data point were used for modeling of the thickness for respective data points. From the grey scale images four different roughness parameters were calculated for each film system.

To gain further knowledge about the surface properties, all cross-linked PEM systems were further characterized by ellipsometry (Figure 5 and Figure 6). The optical analysis of PEMs by ellipsometry is an excellent complementary method to the well-known QCM-D technique. In general, it can be said that the detection of the change in polarization, described by the values ∆ and ρ, allows the calculation of the thickness of thin layers. Furthermore, the determination of both values allows the determination of roughness values from relatively large sections of a sample. The measurements were performed using a spectrum from 300 to 900 nm and confirm, essentially, the layer thicknesses calculated from QCM-D data. As already known from the QCM-D data, the PEM1 system is far thinner than the collagen containing systems PEM2 and PEM3 and a layer thickness of approximately 70 nm was measured (Figure 5). This result confirms both the reliability of the QCM-D data and the validity of the model used for the thickness calculation. The film thicknesses for PEM2 and PEM3 systems were in accordance with the QCM-D data. For both film systems a thickness of about 200–250 nm was determined for the non-cross-linked samples.

However, the ellipsometry data does not allow any conclusion about whether the cross-linking has any impact on the film thickness or the topography of the films. On the contrary, it seems that the film thickness remains nearly unaffected over all EDC concentrations and there is no clear tendency as to whether a higher EDC concentration leads to a swelling or shrinking of the investigated films. The same is true for the roughness of the films. The calculation of the roughness is based on ∆ and ρ maps with a resolution of 1 µm in a scanning area of (531 × 401) µm^2^. With the ∆ and ρ values for each single spot the thickness for each spot can be modeled and subsequently an image can be generated. Next, the image can be used to quantify the roughness of surfaces. In this way, it was possible to demonstrate that EDC cross-linking of the introduced films causes a more or less pronounced variance, or heterogeneity, in the surface roughness (Figure 6). Indeed, it was shown that the PEM3 system causes he most pronounced heterogeneity, especially when the parameters Sa, S10z, and Sdr are considered.

### 3.3. Cellular Response to EDC Cross-Linked PEMs

In a first approach, the initial cellular response to the cross-linked PEM systems was investigated by the measurement of cell proliferation, adhesion, and spreading. The murine pre-osteoblast cell line MC3T3 E1 was used for the analysis, since it is known that the cell line is sensitive to physicochemical stimuli such as substrate stiffness and collagen-binding motif GFOGER by α2β1-integrin expression [21,34,35]. However, it should be noted that EDC cross-linking might inhibit the integrin collagen interaction by blocking of the binding motif due to the reaction of the carboxylate from the glutamic acid within the binding sequence [36]. Therefore, it is then conclusive to speculate that the blocking of the binding motifs at higher EDC concentrations eventually compensates the positive effect of an increasing substrate stiffness regarding the initial cellular response.

**Figure 7:**
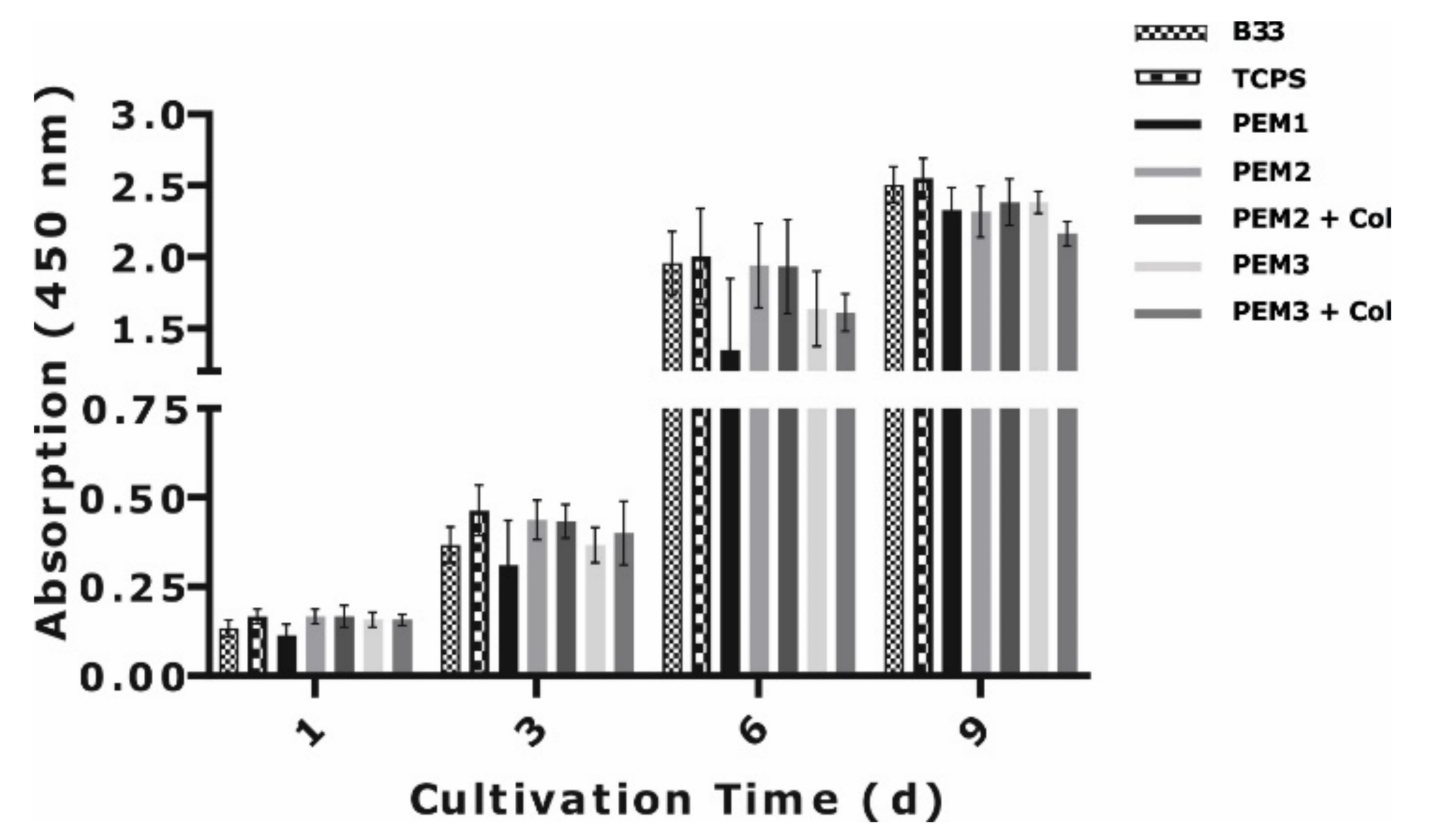
XTT assay for MC3T3 cells cultivated on different PEM films. For PEM2 and PEM3 collagen or Hep was chosen as final cover layer.

In a first experiment, only films without any cross-linking were tested with respect to the proliferation and morphology of osteoblast cells, taking non-modified glass and TCPS as reference (Figure 7). The aim was to find out whether the deposition of collagen as the terminal layer influences the biocompatibility. The performed XTT assay clearly shows no differences between the film systems with collagen or Hep as outermost top layer. Nevertheless, there is the clear tendency that cell proliferation correlates with the collagen content in the films. Hence, lowest proliferation was detected for the collagen-free PEM1 system and the highest for the PEM3 films. This finding demonstrates that the deposition of collagen clearly improves the biocompatibility of the PEMs.

**Figure 8:**
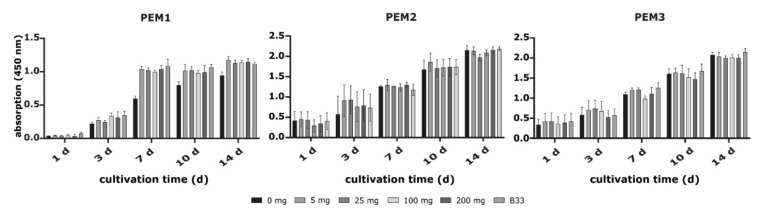
XTT assay for MC3T3 cells cultivated on different PEM films cross-linked with different EDC concentrations.

**Figure 9:**
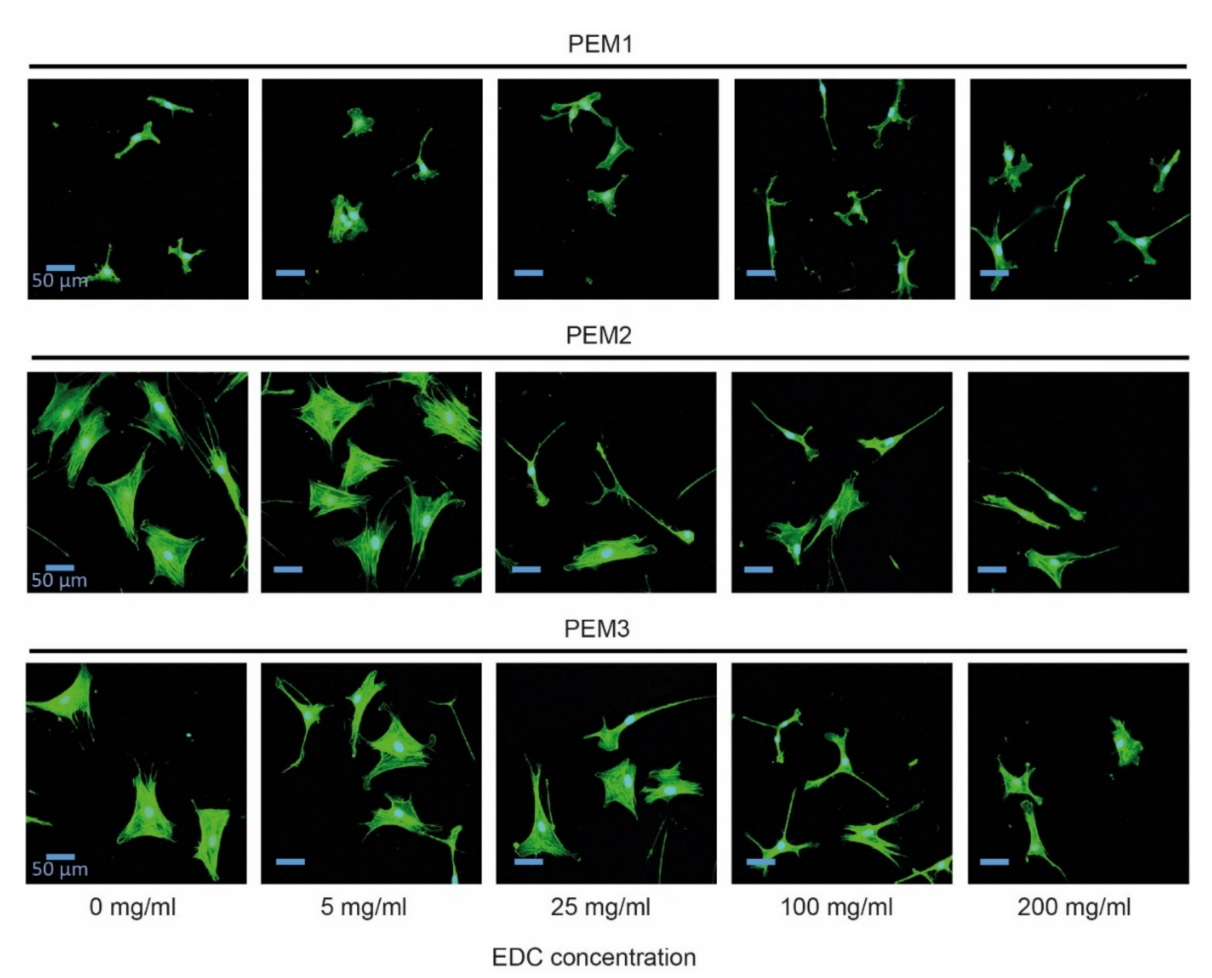
Fluorescence microscope images from MC3T3 cells for analysis of spreading on different PEM films. The experiment was conducted without addition of EDTA to the medium (Scale bar = 50 µm).

**Figure 10:**
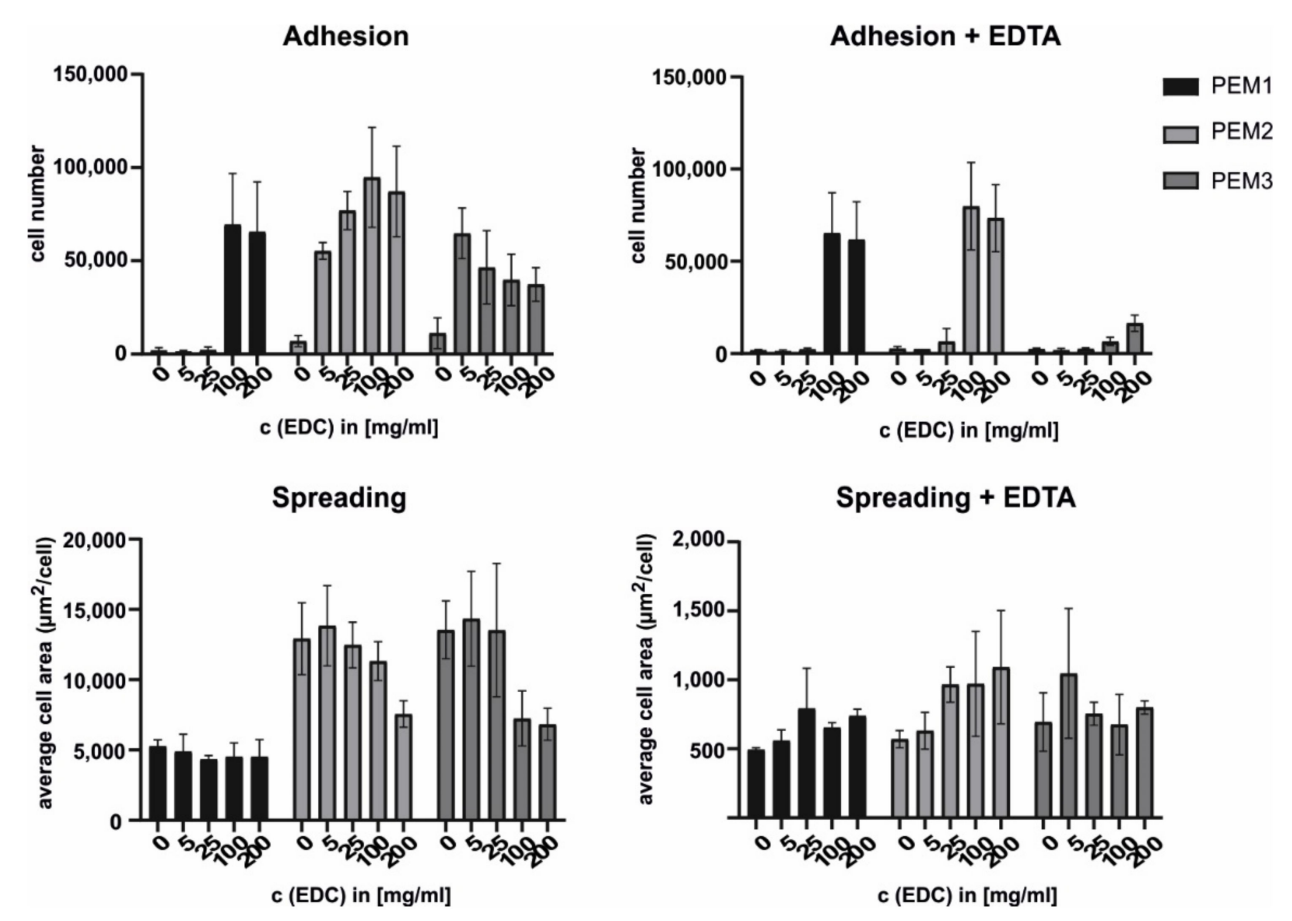
Adhesion and spreading of MC3T3 cells on different PEM films cross-linked with different EDC concentrations. Adhesion and spreading experiments were conducted with or without EDTA in the medium. The EDTA was supplemented in the medium to inhibit integrin-mediated binding to collagen.

Correspondingly, only PEM systems without collagen as the terminal layer were further tested regarding the effect of cross-linking. Again, a proliferation assay was performed (Figure 8) and additionally the adhesion and spreading behavior of the cells was observed (Figure 9 and Figure 10). However, until day three, and also partially day seven, the cell growth is slightly reduced on PEM2 and PEM3 at higher EDC concentrations. On the other hand, EDC cross-linking significantly improves the cell growth on PEM1 films and this effect is even more pronounced for higher EDC concentrations. However, over longer cultivation periods cell growth seems to be only slightly influenced by the cross-linking, due to confluence and contact inhibition of the cell monolayer at higher cell densities.

Further investigations focused on the cell morphology of the MC3T3 E1 cells to confirm the proliferation data. The osteogenic progenitor cells lose their fibroblast cell shape, and the density of the cells seem to decrease on cross-linked PEM2 and PEM3 films with higher EDC concentrations and higher degrees of cross-linking (Figure 9). Again, cross-linking of PEM1 films leads to a significant improvement in the biocompatibility and the cell phenotype on cross-linked films. Obviously, these findings and the knowledge about the impact of EDC/NHS chemistry on integrin binding sites in collagen led to the conclusion that the initial cell contact on collagen films is partially suppressed in correlation with the used EDC/NHS concentration.

A more detailed investigation based on additional adhesion experiments and spreading assays was performed (Figure 10). To allow a conclusive explanation, the adhesion experiments were conducted under conditions with and without EDTA. Briefly, the EDTA complexes all bivalent ions in the solution, such as magnesium ions, and thus inhibits integrin-mediated binding to the surface. The rationale behind this was the identification of integrin-independent cell binding as expected for PEM1 films, since these films do not contain any integrin-specific binding sites. Furthermore, this approach should provide reliable information to what extent the substrate stiffness contributes to cell adhesion and spreading. Therefore, EDTA was added to consider this material-specific stiffness effect on cell adhesion in isolation from the well-known integrin-mediated effect. On PEM1 films, MC3T3 E1 cells adhere most efficiently on films cross-linked with EDC concentrations of 100 and 200 mg/mL. This result could be also reproduced under conditions with EDTA. This strongly confirms the notion that the cell binding in this case is integrin independent and correlates with the multilayer stiffness. By contrast, for PEM3 films with the highest amount of collagen, cell adhesion as well as spreading correlates inversely with the EDC concentration. Without EDTA the number of adhered cells reaches a clear maximum at the lowest EDC concentration of 5 mg/mL, which represents the lowest stiffness but decreases significantly with rising EDC concentration, which represents higher values of film stiffness. The same effect could be observed for the cell spreading without EDTA on PEM2 films. The negative effect of EDC on cell adhesion and spreading was predominantly inverted by the addition of EDTA. After supplementing EDTA, more cells adhere to PEM3 films cross-linked with higher EDC concentrations.

A critical conclusion of these preliminary experiments is that the cross-linking of polyelectrolyte multilayers using EDC/NHS chemistry can adversely affect cell behavior, such as cell adhesion and spreading as a result of the altered availability of the collagen-binding motif, GFOGER. It is recommended to check this carefully in each individual case.

## 4. Discussion

### 4.1. Characteristics of PEM Films

QCM-D data have proven to be an excellent basis for analyzing the growth kinetics of PEMs (Figure 1 and Figure S1). For all multilayer systems the film growth can be characterized by a rather linear increase in thickness, with a gradual transition to an exponential course. The obtained data for PEM1 and PEM2 confirm previous published data and a similar increase in the thicknesses could be observed [32]. A nearly linear growth could be also achieved for the PEM3 film system, the PEM film with the highest number of (Col-Hep) double layers. Thus, with the exception of the first collagen layer, which leads initially to a significant increase in the layer thickness, the following double layers have shown a linear growth kinetic. However, a thorough analysis of the raw data of each single PE layer reveals a significant difference to (PLL-Hep) films. In contrast to (PLL-Hep) double layers there was only a slight increase and even a decrease in frequency shift at lower double layer numbers when Hep was deposited. This observation is comparable with well-known results [37,38,39,40]. In these previous studies the effect was explained by low ionic strength [39], re-dissolution of PE-complexes at the interface between the PEM and the PE-solution [38,39], or swelling/shrinking phenomena after PE adsorption [37]. However, PEM systems containing larger amounts of collagen attain significantly larger thickness values than (PLL-Hep) films.

Collagen represents a weak cation and the QCM-D data, as well as the zeta potential measurements, suggests that there is either a re-dissolution of (Col-Hep) complexes upon deposition of Hep, or a diffusion of Hep molecules into the collagen network, or both. The missing charge (over-)compensation seen in the ζ potential measurements (Figure 2) is obviously a typical consequence of the weak ionic strength of collagen. Thus, deposition of collagen leads to only a slight compensation of the strong negative surface charge induced by heparin. For the first layer there is still a significant increase of the ζ potential, and the surface charge changes from approximately −45 mV to approximately −20 mV. This alternating charge difference decays gradually and reaches a steady state of equilibrium after the deposition of some double layers. A similar reduction of this “oscillation” could be observed in previous studies with collagen and HA as the PE for layer-by-layer deposition [31]. In contrast to the films investigated in the current study, the films with HA showed a clear charge overcompensation. The missing charge reversal for (Col-Hep) films can be explained by the ζ potential measuring conditions at the isoelectric point of collagen (pH 5.5). The pH of the buffer used for the ζ potential measurement (1 mM NaCl; pH 5.5) is higher than the pH from the buffer used for the film construction (pH 3.5) and therefore it is very likely that the collagen molecules change its state of charge after the deposition process [41]. Since it is likely that collagen molecules are not diffusing into the PEM matrix but form a porous network on the surface, it is possible that amino acid residues that are not involved in ionic interactions with heparin molecules will be deprotonated [42]. The deprotonation of collagen and the still accessible heparin molecules led to a negative ζ potential. However, the measured linear growth of PEM3 films by QCM-D confirms that the film growth is based on the alternating deposition of collagen and heparin. Thus, PEM3 films with nine (Col-Hep) double layers reached a film thickness of about 200 nm. These films are significantly thinner than films with (Col-HA) bi-layers, which were described with a thickness of about 270 nm for five double layers. With respect to the strong electrostatic character of heparin, it seems likely that heparin interacts much better with collagen than HA. The sulfate groups of heparin are less hydrated and the interaction in aqueous solutions with NH_3_^+^ groups from proteins is much stronger than the interaction of the COO- groups from the HA with NH_3_^+^ groups [43]. In fact, the lower hydration level and the higher charge density of heparin results in a stronger electrostatic interaction with collagen but lower interactions with free counterions like Na+. This behavior may explain why (Col-Hep) films are thinner than (Col-HA) films. It is comprehensible that (Col-Hep) films like (PLL-Hep) films are just less hydrated than films with other GAGs [32]. This also reflects the in vivo function of the different GAGs. In the ECM functions of HA and CSA are, indeed, directly related to their hydration properties, whereas that of HEP is rather related to its interactions with proteins via its sulfate groups [44,45]. The consideration of different models for the calculation of the layer thickness from QCM-D data (data not shown) confirms a relatively low hydration level. Furthermore, the thickness values of the films could be confirmed by ellipsometry. In general, ellipsometry results were close to the QCM-D results. This confirms not only the reliability of both methods but further indicates that the analyzed films have indeed a low hydration level. In QCM-D measurements, all film components including water molecules are considered, whereas with ellipsometry only an optical contrast can be detected. Essentially, it can be concluded from these data that the interplay between intrinsic charge compensation and extrinsic charge compensation led to different multilayer structures depending on whether the charged polyelectrolyte was balanced by the oppositely charged polyelectrolyte or the charged polyelectrolyte was balanced by the available counterions. The discussion above indicates that the (PLL-Hep) films can be allocated to the first group and the (Col-Hep) films tend to have some properties that indicate at least partial extrinsic charge compensation. It is of value to point out that with increasing extrinsic charge compensation, the polyelectrolyte multilayers become thicker and show a more open structure and more mobile polymer chains. This is exactly what the measurements have shown in this study.

However, the clearly non-linear behavior after the initial deposition of collagen might indicate that there is indeed a massive adsorption of collagen to the surface. However, due to the clear reduction after the first collagen deposition it seems that a significant part of the collagen is only bound in the bulk phase, or diffuses from the layer when it is exposed to the washing solution. The diffusion of collagen molecules can be explained by the pH shift in the washing solution and the reduction of the cationic character at a higher pH. Hence, there is a great influence of extrinsic charge compensation and a diffusion from the surface. Thus, only collagen molecules that are bound to the multilayer by ionic interaction and an intrinsic charge compensation seem to remain at the surface, whereas collagen molecules with no interaction diffuse from the surface. The dissipation from the QCMD (Figure S1) indicates that there is only a low hydration level and thus only a low influence of the bulk phase on the measurement. This further indicates that there is a diffusion of the collagen from surface rather than loosely bound collagen in the bulk phase. It is known that collagen molecules become more insoluble and tend towards self-aggregation at higher pH. This may lead to a higher diffusion of collagen molecules after initial adsorption to the surface. With the deposition of further collagen layers, it seems possible that less collagen binds to the surface, since fewer heparin molecules are directly available at the surface but still prominent within the collagen network. This may explain the negative zeta potential but less collagen binding after the deposition of further collagen layers.

### 4.2. Film Cross-Linking, Physiochemistry, and Topography of PEMs

Film cross-linking with EDC is a well-accepted and investigated method to increase on one hand the stability, and to influence the physiochemical character on the other hand. However, the FTIR analysis of EDC-treated multilayers clearly reveals that the number of amide bonds increase with higher EDC concentrations (Figure 4). EDC/NHS chemistry catalyzes the generation of covalent amide bonds from COO^−^ and NH_3_^+^ groups from heparin and collagen, respectively. It is evident that the reaction works for all cation/anion combinations. Thus, for (PLL-Hep) or (Col-Hep) films newly formed amide bonds were detected after incubation with EDC. That means, even when it is supposed that in (PLL-Hep) films most of the electrostatic interaction is induced between SO_3_^−^/NH_3_^+^ groups, some of the NH_3_^+^ groups will interact with COO^−^ groups with a certain probability. Indeed, it could be shown previously that the proportion of interaction partners of NH_3_^+^ groups in (PLL/Hep) PEM films reflects the molecule stoichiometry from COO^−^ versus SO_3_^−^ app. 2.5:1 in heparin [31]. The efficacy of the cross-linking of collagen with EDC/NHS has also been shown previously in several publications [36,46,47,48,49,50]. Nevertheless, only few publications are available where layer-by-layer techniques were used for the deposition of substrates [31,42,51] and only a few authors have subsequently used EDC/NHS for cross-linking. However, in the current work it could be shown for the first time that (Col-Hep) films can be cross-linked with EDC/NHS and that the multilayer properties are related to the EDC concentration (Figure 3 and Figure 4).

As for PEM1 films, for PEM2 and PEM3 films the proportion of amino bonds increases with higher EDC concentrations (Figure 3). As expected, an increase in the degree of cross-linking leads to an increase in the Young’s modulus of respective multilayers (Figure 4). The AFM measurements reveal that even the lowest EDC concentration of 5 mg/mL changes the stiffness of the multilayers significantly. Within the used EDC concentration range the stiffness could be adapted in a range between 5 kPa and 650 kPa. Thus, it becomes clear that EDC can be used to create PEM with individually tailored biomechanical properties. This is essential to improve osseointegration of implants. It is generally accepted that films with higher stiffness increase the biocompatibility for bone cells.

It must be emphasized in this context that the Young’s moduli calculated here are to be understood as system parameters and not as material parameters. They are used for benchmarking the layer systems against each other and for measuring the influence of post-crosslinking.

The Hertzian model used is based on homogeneous, isotropic, linear elastic assumptions, which a PEM coating does not possess. Rather, a PEM is characterized by viscoelastic behavior, which was not taken into account here. The published data should therefore be understood as a benchmark among the individual coating systems in the sense of an “apparent modulus” and not as a physical material constant. We recently published a novel evaluation methodology for viscoelastic characterization of fast relaxing hydrogels based on relaxation measurements [52]. In current work, we are adapting this method for relaxation measurements using AFM on thin film coatings in order to be able to study the cellular response to mechanical properties from a viscoelastic point of view.

However, it seems that EDC cross-linking of collagen-rich PEM3 multilayers causes an increase in layer thickness and obviously a less compact layer architecture induced by the weaker intrinsic charge compensation. Topography data from (Col-Hep) multilayers are comparable to those from (Col-HA) films [31,42]. However, that the topographic features on some samples are mainly due to collagen deposition [31] can be concluded from the finding that PEM1 multilayers, as other PLL-GAG multilayers, have almost homogeneous flat surfaces, whereas collagen-rich PEM3 multilayers exhibit a strong increase in layer thickness and heterogeneous topographic structures (Figure 6 and Figure S6). A similar result was obtained from AFM measurements (Figure S2). This can be explained by the fact that collagen is producing fibrillar structures and as such it is only a weak polyelectrolyte, as shown in the zeta potential measurements (Figure 2). Hence, collagen molecules can associate in more loosely bound network structures to the surface of PEM films.

In general, one must note that the roughness of the analyzed films and other polysaccharide-based films is quite low in comparison with other materials like etched and sandblasted titanium (data not shown). Ren and colleagues have already shown that such small roughness values with only slight changes after EDC treatment have only minor effects on cell behavior. Beside topography, they also investigated the surface chemistry, wettability, and stiffness of (PLL/HA) cross-linked films. It was concluded that stiffness is by far the most important parameter [18].

### 4.3. Impact of Cross-Linking on Cellular Response

The effect of cross-linking on cellular behavior was investigated by the analysis of proliferation, adhesion and spreading of MC3T3 cells seeded on the surface of the polyelectrolyte multilayers. Initially the proliferation on uncross-linked films was tested (Figure 8). It became obvious that until day six the cells proliferated much better when collagen was used as a weak cation during layer formation, and respective PEMs reach a proliferation rate similar to the common reference surfaces (B33 glass and TCPS). This is a strong indicator that the incorporation of collagen clearly improves the biocompatibility, almost to the level of cell culture materials. Another central finding from this experiment is the fact that there is no significant difference between PEMs with collagen or heparin when applied as the outermost top layer. This can be explained by the circumstance that collagen layers consist of a porous network rather than a compact homogeneous layer and heparin molecules diffuse into the network [42,53]. The consequence of this molecular behavior is that heparin induces changes in the surface charge and growth kinetic of the multilayer, but at the surface, collagen molecules and binding motifs are still available. The hypothesis is further underlined by the surface analysis with ellipsometry.

Another focus of the cell experiments was the effect of cross-linking on cellular processes, particularly on proliferation, cell adhesion, and spreading [20,21,54]. However, it is also well-described that collagen type I contains specific integrin recognition sites with the amino acid sequence (GFOGER) [55]. This motif mediates the specific binding of β1-containing integrins like α1β1, α2β2, α10β1, and α11β1. The sequence contains the amino acid side chain of glutamate with a primary carboxylate group. However, EDC/NHS treatment consumes the carboxylate groups and the binding motif becomes dysfunctional [36,56]. In general, the data from the current study confirms the effects of EDC/NHS cross-linking on cellular behavior. This includes changes in the multilayer stiffness as well as the availability of integrin binding sites in collagen molecules. The analysis of the proliferation is not significant and shows only a tendency (Figure 8). Hence, for the collagen films PEM2 and PEM3, the proliferation decreases slightly with increasing EDC concentration, whereas for PEM1 films cross-linking clearly shows a positive effect. The increasing proliferation of MC3T3 cells on cross-linked PEM1 films can be justified by the mechanical changes induced by the EDC treatment. As for other cells [20,57,58], MC3T3 E1 cells are specifically sensitive to substrate stiffness and, due to their origin from an osteoblastic lineage, stiffer substrates are preferred [21,59]. However, the results from PEM2 and PEM3 suggest that the cells are also sensitive for the collagen binding sites and the slight decrease in proliferation indicates that the consumption of the binding sites by EDC/NHS cross-linking has a negative effect on cellular behavior. The compensation of the effect after longer incubation is probably mainly due to cell contact inhibition (confluence) and the buildup of a natural ECM by the cells. The interplay between material stiffness and availability of functional binding sites becomes more visible by the analysis of the initial cell adhesion and the spreading (Figure 10, Figures S4 and S5). The visual analysis of the cells clearly indicates a positive effect for collagen integration that is reversed by EDC treatment. Thus, the cells have a more spread phenotype on PEM2 and PEM3 than on PEM1. But the cell area of MC3T3 cell decreases significantly on collagen-containing films cross-linked with EDC (Figure 9). This finding can be also confirmed by a quantitative analysis of the cell area (Figure 10). In contrast to the results for PEM2 and PEM3, no changes were observed for PEM1 films. This finding is a clear hint that not the integrins, but Mg^2+^-independent receptors seem to mediate MC3T3 spreading on a PEM1 film surface. In contrast to this, it is obvious that EDC treatment influences the initial adhesion process of MC3T3 cells to PEM1 films. The strong increase in adhesion to PEM1 films treated with 100 or 200 mg/mL EDC can be interpretated as a stiffness effect, since the same was be observed for PEM2 films, where the change of the stiffness could be measured with AFM. Interestingly, both film systems show a similar character within EDTA-containing medium. This might be due to the similar molecular composition of the PEMs and would explain why the adhesion on PEM2 films increases despite treatment with higher EDC concentrations, whereas the adhesion on PEM3 films is strongly reduced. Indeed, it seems that the loss of integrin binding sites can be compensated by the character of highly cross-linked (PLL-Hep) multilayers. The ability of cell adhesion on cross-linked PEM films without any need of additional adhesive protein recognition sites has been shown before [18]. However, the exact mechanism behind the massive increase needs to be further investigated. Considering the data, it is likely that there exists a stiffness threshold for effective adhesion to the surface. Furthermore, there is a complex interplay between matrix rigidity and the availability of specific binding sites. The balance between these two parameters finally regulates the cellular behavior.

## 5. Conclusions

The motivation of this study was the generation of a flexible PEM platform system that meets the requirements for any purpose in bone contact applications. A bone compatible surface should be achieved by the integration of site-specific ECM molecules and subsequent cross-linking of these molecules to achieve rigid films with a bone like appearance. To reach this goal, for the first-time (Col-Hep) films and (PLL-Hep)–(Col-Hep) hybrid film systems were fabricated and cross-linked with EDC/NHS chemistry. The native films were fully characterized regarding the multilayer growth kinetics in comparison with a (PLL-Hep) reference. Analysis of film growth reveals a nearly linear growth for all PEM systems and topographical analysis confirms the special position of collagen-rich multilayers (PEM3) after EDC cross-linking. The efficacy of the cross-linking was confirmed by FTIR measurements, and the analysis reveals that the quantity of newly created amide bonds correlates with the used EDC concentration. Thus, it was possible to generate PEM with an individual physicochemical character. The change in the film stiffness was analyzed with AFM and a positive correlation with the EDC concentration was documented. Preliminary cellular analysis with the pre-osteoblast murine cell line MC3T3, demonstrated that deposition of collagen significantly improves the biocompatibility and EDC/NHS cross-linking changes the cellular behavior. The cellular response depends on the EDC concentration. Nevertheless, more cellular investigations are needed to identify the full potential of the new polyelectrolyte multilayer systems based on collagen and heparin. Furthermore, it is intended to test these ultrathin films as drug delivery systems. Collagen and heparin are both natural molecules of the ECM with distinct functions regarding cell matrix interaction and immobilization of soluble matrix components such as cytokines. These properties render these molecules, and multilayers made thereof, as ideal candidates for therapeutical applications in bone regeneration or tissue remodeling applications.

## Data Availability

Not applicable.

## Supplementary Materials

The following supporting information can be downloaded at: https://www.mdpi.com/article/10.3390/nano12122054/s1, Figure S1: Online measurement of PEM film growth followed by in situ QCM-D. Shown are differences in the QCM frequency and dissipation shifts (-∆f/v and ppm) as a function of polyelectrolyte layer deposition. Brackets show the number of the accumulated bi-layers deposited on the QCM-D-sensor Data are given for three harmonics: 15 MHz (ƒ); 25 MHz (F); and 35 MHz. Mean values are from at least three independent experiments. Each data point was calculated as mean from 10 values at the end of the deposition cycle. Figure S2: (a) Representative AFM images of all PEM coatings investigated in the manuscript and (b) summarizing data from the topographical analysis. Figure S3: Representative IR spectra of cross-linked film systems. Shown are peaks between 1800 and 1450 nm (Amid I between 1690–1620 cm^−1^ and Amid II between 1590 and 1520 cm^−1^). For better visualization, the graphs were normalized and the bottom line corrected. Thus, the shown graphs are not directly comparable with data in Figure 3, since the analysis was conducted with unprocessed data. Figure S4: Fluorescence microscope images from MC3T3 cells after performing an adhesion experiment on different PEM films without EDTA in the medium. Figure S5: Fluorescence microscope images from MC3T3 cells after performing an adhesion experiment on different PEM films with EDTA in the medium. Figure S6: Optical ellipsometry grey-scale images. Table S1: (a,b) Statistical analysis of the results from Figure 10. For the analysis an unpaired *t*-test with a normality test (Shapiro–Wilk test) was performed. As significance value the two-tailed *p*-value is shown. The calculation of the significance was conducted with SigmaPlot 14.0.
