# Supplementary material for: Tailored Polyelectrolyte Multilayer Systems by Variation of Polyelectrolyte Composition and EDC/NHS Cross-Linking: Physicochemical Characterization and In Vitro Evaluation
Source: Nanomaterials (Basel). 2022 Jun 15;12(12):2054. doi: 10.3390/nano12122054 (PMC9228333; doi:10.3390/nano12122054)
Supplement: Supplementary file 1 [file nanomaterials-12-02054-s001.zip › nanomaterials-1721951-supplementary.pdf]

nanomaterials

MDPI

Supplementary Material

Tailored Polyelectrolyte Multilayer Systems by Variation of Polyelectrolyte Composition and EDC/NHS Cross-Linking: Physicochemical Characterization and in Vitro Evaluation

Uwe Schirmer [1], Johanna Ludolph [1], Holger Rothe [1], Nicole Hauptmann [1], Christina Behrens [2], Eva Bittrich [3], Hen-ning Schliephake [2] and Klaus Liefeith [1,*]

[1] Institute for Bioprocessing and Analytical Measurement Techniques, Heiligenstadt, Germany; uwe.schirmer@iba-heiligenstadt.de (U.S.); johanna.ludolph@t-online.de (J.L.); holger.rothe@iba-heiligenstadt.de (H.R.); nicole.hauptmann@iba-heiligenstadt.de (N.H.)

[2] Department of Oral and Maxillofacial Surgery, George-Augusta-University, Göttingen, Germany; cbehren@gwdg.de (C.B.); schliephake.henning@med.uni-goettingen.de (H.S.)

[3] Center Macromolecular Structure Analysis, Leibniz Institute of Polymer Research, Dresden, Germany; bittrich-eva@ipfdd.de

* Correspondence: Klaus.Liefeith@iba-heiligenstadt.de; Tel.: +49-3606-671500

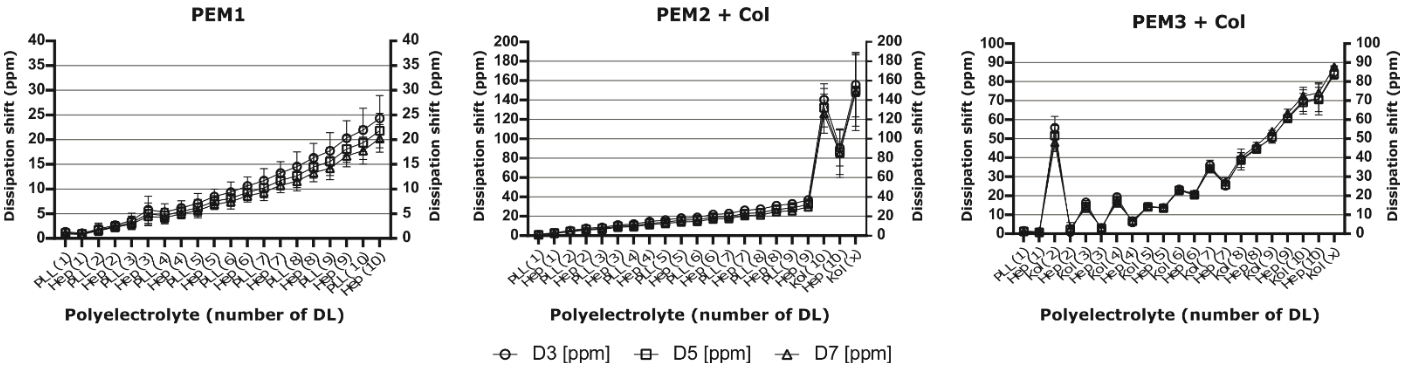

Figure S1. Online measurement of PEM film growth followed by in situ QCM-D. Shown are differences in the QCM frequency and dissipation shifts (-Δf/v and ppm) as a function of polyelectrolyte layer deposition. Brackets show the number of the accumulated bi-layers deposited on the QCM-D-sensor Data are given for three harmonics: 15 MHz (*f*); 25 MHz (F); and 35 MHz. Mean values are from at least three independent experiments. Each data point was calculated as mean from 10 values at the end of the deposition cycle.

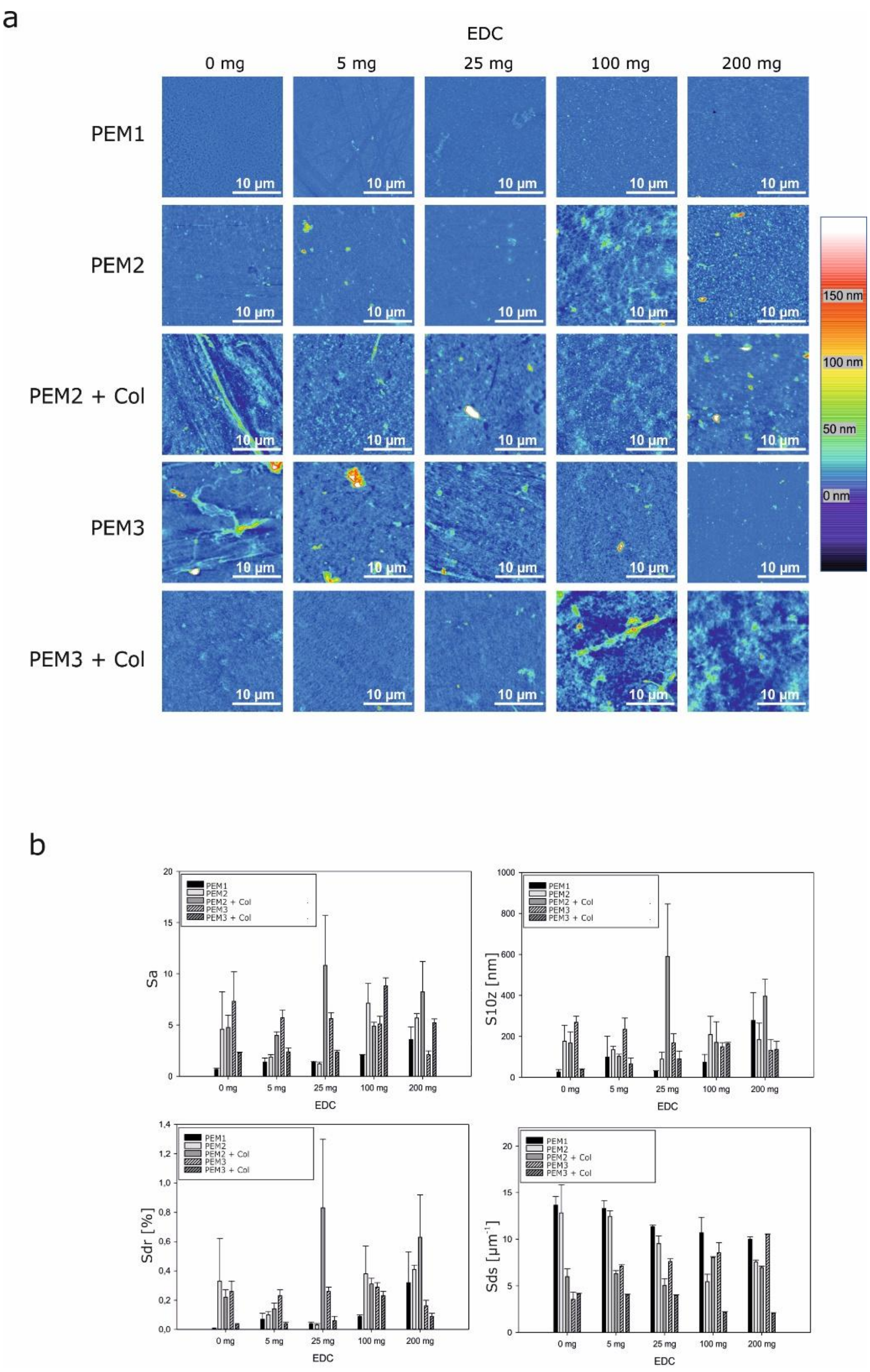

Figure S2. (**a**) Representative AFM images of all PEM coatings investigated in the manuscript and (**b**) summarizing data from the topographical analysis.

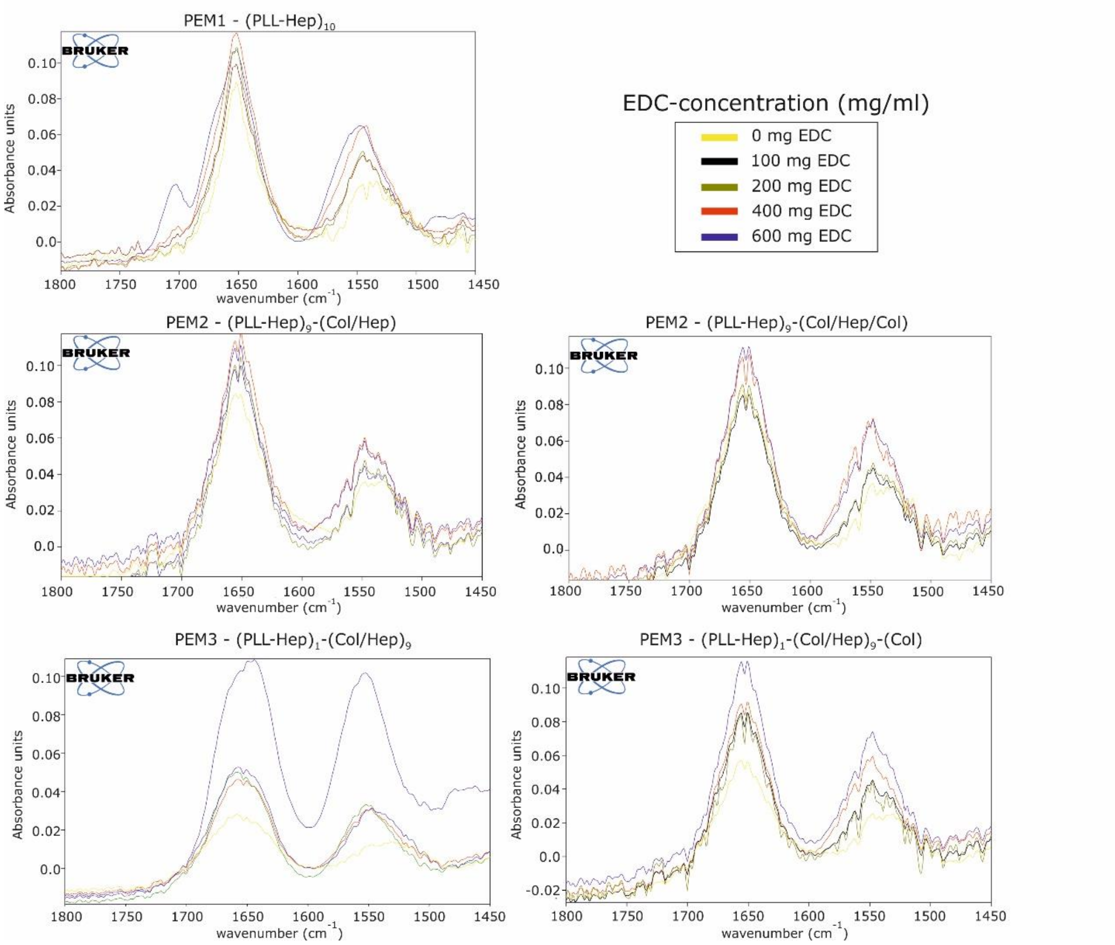

Figure S3. Representative IR spectra of cross-linked film systems. Shown are peaks between 1800 and 1450 nm (Amid I between 1690–1620 cm^{-1} and Amid II between 1590 and 1520 cm^{-1}). For better visualization, the graphs were normalized and the bottom line corrected. Thus, the shown graphs are not directly comparable with data in Figure 3, since the analysis was conducted with unprocessed data.

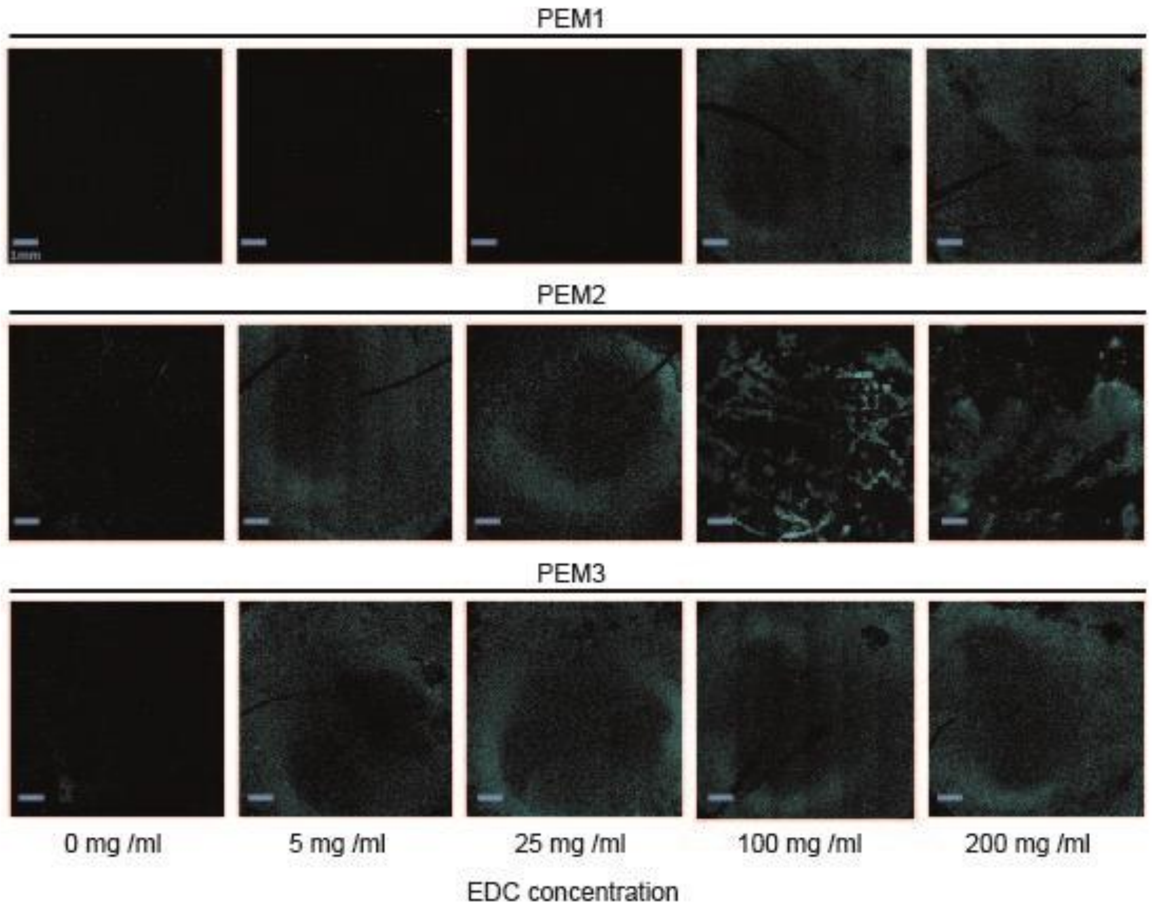

Figure S4. Fluorescence microscope images from MC3T3 cells after performing an adhesion experiment on different PEM films without EDTA in the medium.

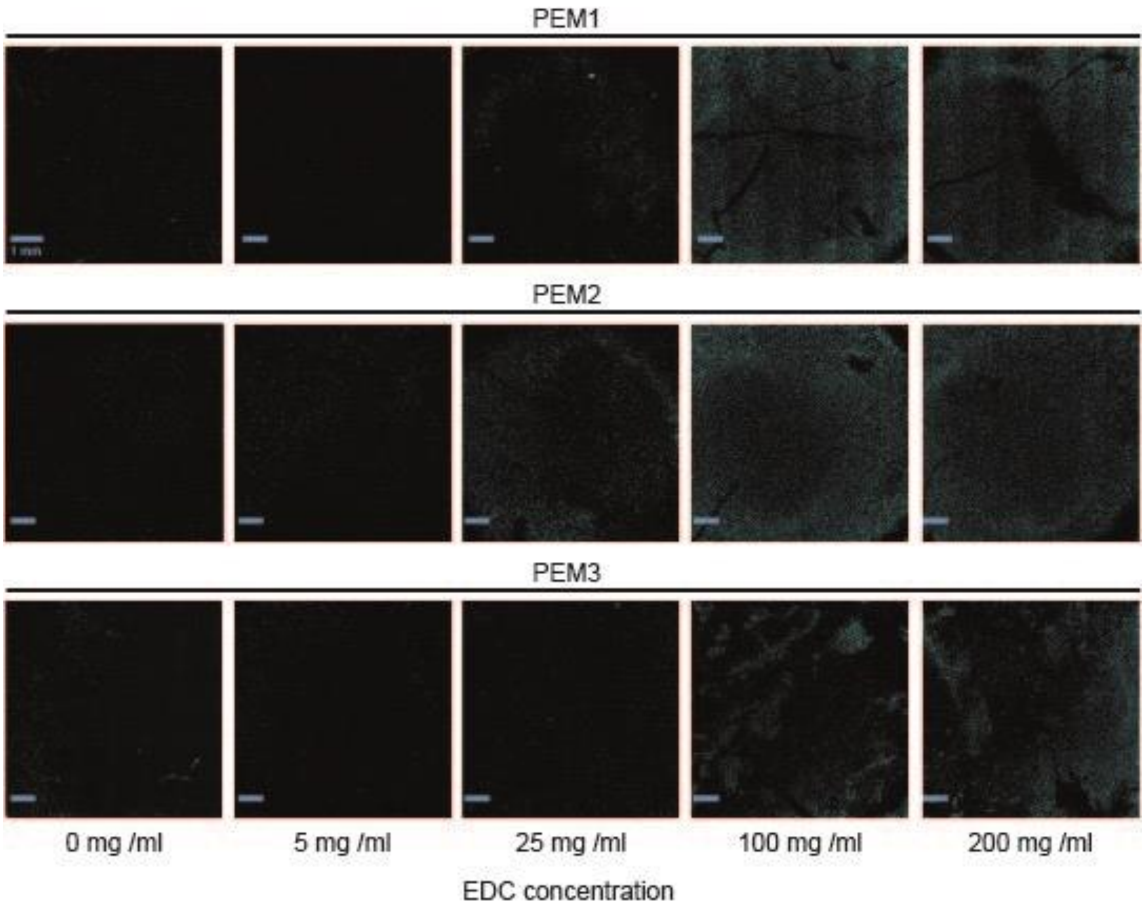

Figure S5. Fluorescence microscope images from MC3T3 cells after performing an adhesion experiment on different PEM films with EDTA in the medium.

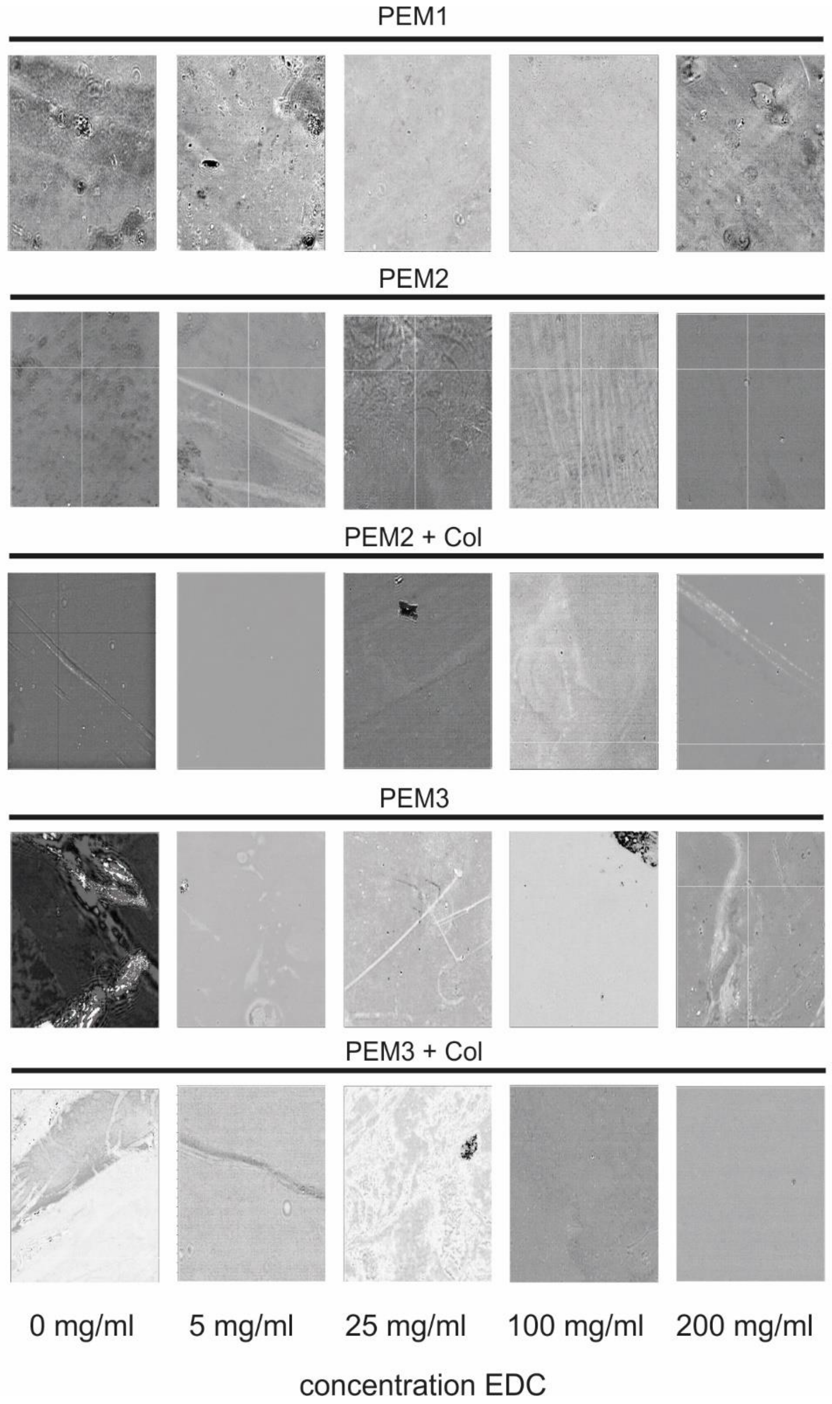

Figure S6. Optical ellipsometry grey-scale images.

Adhesion

	without EDTA					with EDTA			
	Compared samples	normality Test (Shapiro-Wilk)	two-tailed P-value (t-test)			Compared samples	normality Test (Shapiro-Wilk)	two-tailed P-value (t-test)	
PEM1	0 mg vs 5 mg	passed	0.551	n.s.	PEM1	0 mg vs 5 mg	passed	0.3	n.s.
	0 mg vs 25 mg	passed	0.79	n.s.		0 mg vs 25 mg	passed	0.331	n.s.
	0 mg vs 100 mg	passed	0.013	*		0 mg vs 100 mg	passed	0.00737	**
	0 mg vs 200 mg	passed	0.0145	*		0 mg vs 200 mg	passed	0.00724	**
	5 mg vs 25 mg	passed	0.381	n.s.		5 mg vs 25 mg	passed	0.381	n.s.
	5 mg vs 100 mg	passed	0.0133	*		5 mg vs 100 mg	passed	0.0133	*
	5 mg vs 200 mg	passed	0.014	*		5 mg vs 200 mg	passed	0.014	*
	25 mg vs 100 mg	passed	0.0133	*		25 mg vs 100 mg	passed	0.0133	*
	25 mg vs 200 mg	passed	0.0148	*		25 mg vs 200 mg	passed	0.0148	*
	100 mg vs 200 mg	passed	0.871	n.s.		100 mg vs 200 mg	passed	0.848	n.s.
PEM2	0 mg vs 5 mg	passed	0.00623	**	PEM2	0 mg vs 5 mg	passed	0.307	n.s.
	0 mg vs 25 mg	passed	0.0115	*		0 mg vs 25 mg	passed	0.489	n.s.
	0 mg vs 100 mg	passed	0.0442	*		0 mg vs 100 mg	passed	0.00874	**
	0 mg vs 200 mg	passed	0.0435	*		0 mg vs 200 mg	passed	0.00533	**
	5 mg vs 25 mg	passed	0.111	n.s.		5 mg vs 25 mg	passed	0.364	n.s.
	5 mg vs 100 mg	passed	0.177	n.s.		5 mg vs 100 mg	passed	0.00845	**
	5 mg vs 200 mg	passed	0.209	n.s.		5 mg vs 200 mg	passed	0.00512	**
	25 mg vs 100 mg	passed	0.473	n.s.		25 mg vs 100 mg	passed	0.0108	*
	25 mg vs 200 mg	passed	0.638	n.s.		25 mg vs 200 mg	passed	0.00726	**
	100 mg vs 200 mg	passed	0.795	n.s.		100 mg vs 200 mg	passed	0.79	n.s.
PEM3	0 mg vs 5 mg	passed	0.00424	**	PEM3	0 mg vs 5 mg	passed	0.913	n.s.
	0 mg vs 25 mg	passed	0.0457	*		0 mg vs 25 mg	passed	0.308	n.s.
	0 mg vs 100 mg	passed	0.132	n.s.		0 mg vs 100 mg	passed	0.0353	*
	0 mg vs 200 mg	passed	0.0347	*		0 mg vs 200 mg	passed	0.00875	**
	5 mg vs 25 mg	passed	0.256	n.s.		5 mg vs 25 mg	passed	0.361	n.s.
	5 mg vs 100 mg	passed	0.0297	*		5 mg vs 100 mg	passed	0.0391	*
	5 mg vs 200 mg	passed	0.058	n.s.		5 mg vs 200 mg	passed	0.00903	**
	25 mg vs 100 mg	passed	0.382	n.s.		25 mg vs 100 mg	passed	0.0108	*
	25 mg vs 200 mg	passed	0.361	n.s.		25 mg vs 200 mg	passed	0.00726	**
	100 mg vs 200 mg	passed	0.881	n.s.		100 mg vs 200 mg	passed	0.104	n.s.

Spreading

	without EDTA					with EDTA			
	Compared samples	normality Test (Shapiro-Wilk)	two-tailed P-value (t-test)			Compared samples	normality Test (Shapiro-Wilk)	two-tailed P-value (t-test)	
PEM1	0 mg vs 5 mg	passed	0.643	n.s	PEM1	0 mg vs 5 mg	passed	0.368	n.s
	0 mg vs 25 mg	passed	0.0379	*		0 mg vs 25 mg	passed	0.284	n.s
	0 mg vs 100 mg	passed	0.301	n.s.		0 mg vs 100 mg	passed	0.0263	*
	0 mg vs 200 mg	passed	0.378	n.s.		0 mg vs 200 mg	passed	0.02	*
	5 mg vs 25 mg	passed	0.497	n.s.		5 mg vs 25 mg	passed	0.387	n.s
	5 mg vs 100 mg	passed	0.713	n.s.		5 mg vs 100 mg	passed	0.258	n.s
	5 mg vs 200 mg	passed	0.737	n.s.		5 mg vs 200 mg	passed	0.109	n.s
	25 mg vs 100 mg	passed	0.767	n.s		25 mg vs 100 mg	passed	0.572	n.s
	25 mg vs 200 mg	passed	0.81	n.s		25 mg vs 200 mg	passed	0.823	n.s
	100 mg vs 200 mg	passed	0.999	n.s.		100 mg vs 200 mg	passed	0.179	n.s
PEM2	0 mg vs 5 mg	passed	0.699	n.s	PEM2	0 mg vs 5 mg	passed	0.622	n.s
	0 mg vs 25 mg	passed	0.81	n.s		0 mg vs 25 mg	passed	0.0591	n.s
	0 mg vs 100 mg	passed	0.396	n.s		0 mg vs 100 mg	passed	0.279	n.s
	0 mg vs 200 mg	passed	0.0271	*		0 mg vs 200 mg	passed	0.218	n.s
	5 mg vs 25 mg	passed	0.511	n.s		5 mg vs 25 mg	failed	0.355	n.s
	5 mg vs 100 mg	passed	0.242	n.s		5 mg vs 100 mg	passed	0.271	n.s
	5 mg vs 200 mg	passed	0.0224	*		5 mg vs 200 mg	passed	0.988	n.s
	25 mg vs 100 mg	passed	0.406	n.s		25 mg vs 100 mg	passed	0.988	n.s
	25 mg vs 200 mg	passed	0.0107	*		25 mg vs 200 mg	passed	0.72	n.s
	100 mg vs 200 mg	passed	0.0174	n.s		100 mg vs 200 mg	passed	0.789	n.s
PEM3	0 mg vs 5 mg	passed	0.747	n.s	PEM3	0 mg vs 5 mg	passed	0.514	n.s
	0 mg vs 25 mg	passed	0.995	n.s		0 mg vs 25 mg	passed	0.743	n.s
	0 mg vs 100 mg	passed	0.0185	*		0 mg vs 100 mg	failed		n.s
	0 mg vs 200 mg	passed	0.00781	**		0 mg vs 200 mg	passed	0.559	n.s
	5 mg vs 25 mg	passed	0.822	n.s		5 mg vs 25 mg	passed	0.479	n.s
	5 mg vs 100 mg	passed	0.0345	*		5 mg vs 100 mg	passed	0.419	n.s
	5 mg vs 200 mg	passed	0.0217	n.s		5 mg vs 200 mg	passed	0.539	n.s
	25 mg vs 100 mg	passed	0.101	n.s		25 mg vs 100 mg	passed	0.68	n.s
	25 mg vs 200 mg	passed	0.0761	n.s		25 mg vs 200 mg	passed	0.567	n.s
	100 mg vs 200 mg	passed	0.765	n.s		100 mg vs 200 mg	passed	0.514	n.s

Table S1. a and b: Statistical analysis of the results from Figure 10. For the analysis an unpaired *t*-test with a normality test (Shapiro–Wilk test) was performed. As significance value the two-tailed *p*-value is shown. The calculation of the significance was conducted with SigmaPlot 14.0.
